# Group A Streptococcal S Protein Utilizes Red Blood Cells as Immune Camouflage and Is a Critical Determinant for Immune Evasion

**DOI:** 10.1016/j.celrep.2019.11.001

**Published:** 2019-12-03

**Authors:** Igor H. Wierzbicki, Anaamika Campeau, Diana Dehaini, Maya Holay, Xiaoli Wei, Trever Greene, Man Ying, Jenna S. Sands, Anne Lamsa, Elina Zuniga, Kit Pogliano, Ronnie H. Fang, Christopher N. LaRock, Liangfang Zhang, David J. Gonzalez

**Affiliations:** 1 Department of Pharmacology and the Skaggs School of Pharmacy and Pharmaceutical Sciences, University of California, San Diego, La Jolla, CA 92093, USA; 2 Department of NanoEngineering and Chemical Engineering Program, University of California, San Diego, La Jolla, CA 92093, USA; 3 Department of Biology, University of California, San Diego, La Jolla, CA 92093, USA; 4 Department of Microbiology and Immunology, Division of Infectious Diseases, and Antimicrobial Resistance Center, Emory University, Atlanta, GA 30322, USA; 5 Department of Biological Sciences, University of California, San Diego, La Jolla, CA 92037, USA; 6 These authors contributed equally; 7 Lead Contact

## Abstract

Group A *Streptococcus* (GAS) is a human-specific pathogen that evades the host immune response through the elaboration of multiple virulence factors. Although many of these factors have been studied, numerous proteins encoded by the GAS genome are of unknown function. Herein, we characterize a biomimetic red blood cell (RBC)-captured protein of unknown function—annotated subsequently as S protein—in GAS pathophysiology. S protein maintains the hydrophobic properties of GAS, and its absence reduces survival in human blood. S protein facilitates GAS coating with lysed RBCs to promote molecular mimicry, which increases virulence *in vitro* and *in vivo*. Proteomic profiling reveals that the removal of S protein from GAS alters cellular and extracellular protein landscapes and is accompanied by a decrease in the abundance of several key GAS virulence determinants. *In vivo*, the absence of S protein results in a striking attenuation of virulence and promotes a robust immune response and immunological memory.

## In Brief

Wierzbicki et al. show that S protein is a major group A Streptococcus (GAS) virulence factor that facilitates bacterial coating with lysed red blood cells to promote molecular mimicry, which increases virulence *in vitro* and *in vivo*. Removal of S protein reduces the abundance of multiple virulence factors and attenuates virulence.

## Graphical Abstract

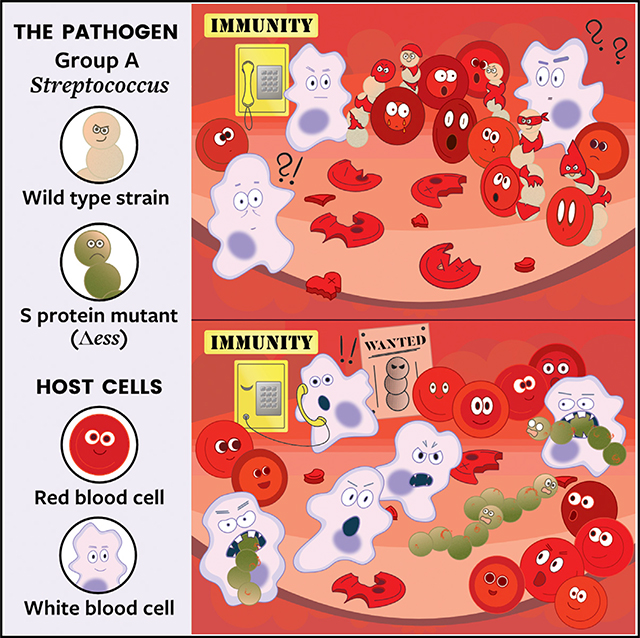

## INTRODUCTION

*Streptococcus pyogenes* (group A *Streptococcus* [GAS]) is a leading health and economic burden worldwide, with an estimated 700 million infections occurring annually. Among these are 18.1 million severe cases that result in over 500,000 deaths (Carapetis et al., 2005). Despite active research, a protective vaccine remains elusive (Dale et al., 2016; Rivera-Hernandez et al., 2016), leaving antimicrobial agents as the sole pharmacological intervention against GAS. To date, penicillin remains a primary drug of choice for combating GAS infections. However, despite no apparent emergence of resistant isolates, the rate of treatment failures with penicillin has increased to nearly 40% in certain regions of the world (Brook, 2013). Due to the high prevalence of GAS infection and the decreasing efficacy of the available repertoire of countermeasures, it is critical to investigate alternative approaches against GAS infection.

An emerging strategy for combating drug-resistant bacteria involves targeting virulence (Allen et al., 2014; Baron, 2010; Rasko and Sperandio, 2010). GAS has evolved to readily colonize and thrive within the human host. To avoid immune clearance, GAS expresses a wide variety of secreted and cell-associated virulence factors to facilitate survival during infection. The pantheon of these virulence factors is extensive, and some such as Streptolysin S have been studied for over 100 years (Molloy et al., 2011). Despite decades of inquiry into the role and regulation of GAS virulence factors, the function and potential importance of many proteins involved in pathogenicity remain unknown.

To facilitate the exploration of virulence factors, our group recently developed Biomimetic Virulomics, a tool that uses nanotechnology-enabled affinity enrichment coupled with multiplexed quantitative proteomics. This tool successfully enriched red blood cell (RBC)-specific effector proteins secreted by GAS (Distler and Tenzer, 2017; Lapek et al., 2017a). Among the identified proteins were known blood toxins, such as Streptolysin O and CAMP factor. Also identified were several proteins of unknown function.

Here, we examine one of the previously uncharacterized proteins captured by our RBC-based Biomimetic Virulomics study, SPy_0802 (henceforth named S protein), and investigate its role in GAS pathogenesis. Using an in-frame deletion mutant, Δ*ess*, we characterized the impact of S protein on GAS physiology, its interactions with human RBCs and phagocytic cells *in vitro*, its role in cellular and extracellular proteome composition, and its virulence in an *in vivo* murine model of disseminated infection. Furthermore, we examined host responses through quantitative proteomic analysis of splenic tissues infected with wild-type (WT) GAS or the Δ*ess* mutant. Because of its pivotal roles in pathogenesis and immune evasion and its conserved nature in *Streptococci*, S protein shows promising clinical potential as a target for the development of anti-virulence pharmacological interventions.

## RESULTS

### S Protein Is a Highly Conserved Protein across GAS Serotypes

**Figure 1.**
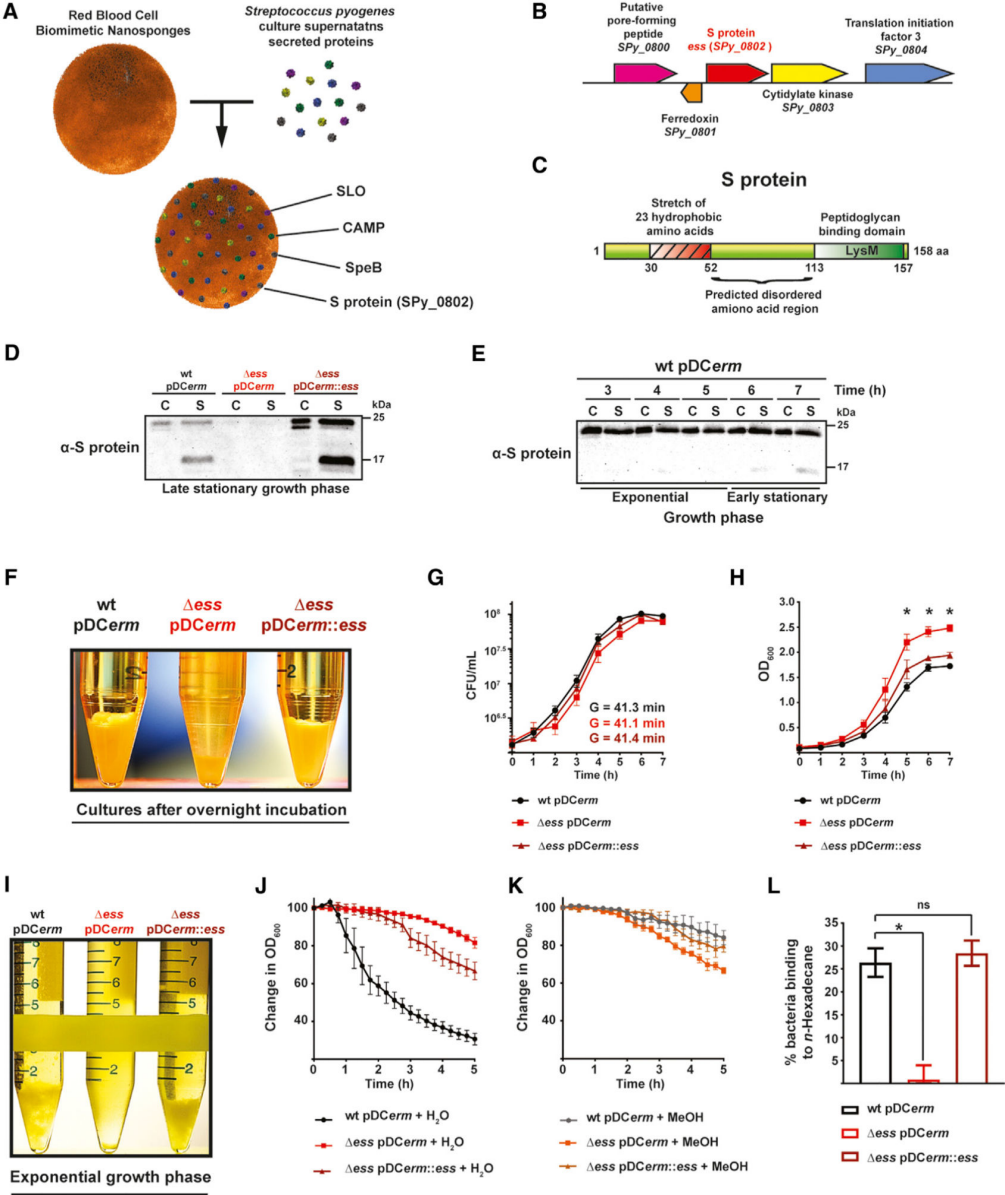
S Protein Discovery and Initial Characterization (A) Schematic of S protein discovery by Biomimetic Virulomics. (B) Localization of *ess* (*SPy_0802*) and surrounding genes. (C) Predicted protein architecture of S protein. (D) α-S protein antiserum validation of cells (Cs) and supernatants (Ss). (E) Western blotting analysis of S protein secretion and processing in Cs and Ss during growth of wild-type (WT) GAS. (F) Photographic documentation of GAS sedimentation after overnight growth. (G) Proliferation of GAS strains in standard growth medium (G, bacterial generation time), in biological triplicate with data as mean ± SEM (*p < 0.05). (H) Proliferation of GAS strains in standard growth medium, in biological triplicate with data as mean ± SEM (*p < 0.05). (I) Photographic documentation of culture sedimentation during exponential phase. (J) Sedimentation of GAS overnight cultures mixed with water measured as a change in optical density 600 (OD_600_) over 5 h, in biological triplicate with data as mean ± SEM (*p < 0.05). (K) Sedimentation of GAS overnight cultures mixed with methanol measured as a change in OD_600_ over 5 h, in biological triplicate with data as mean ± SEM (*p < 0.05). (L) Measurement of bacterial cells hydrophobic properties, in biological triplicate with data as mean ± SEM (*p < 0.05).

SPy_0802 was identified through the use of Biomimetic Virulomics, a recently published affinity-capture method that uses mass-spectrometry-based proteomics of RBC-coated nanoparticles incubated with GAS culture supernatants (Figure 1A) (Lapek et al., 2017a). The *SPy_0802* locus is highly conserved and largely specific among the members of the *Streptococcus* genus (Figure 1B). SPy_0802 is a relatively small protein composed of an N-terminal hydrophobic region and a C-terminal peptidoglycan-binding motif, LysM (Figure 1C). The alignment of SPy_0802 sequences from 20 available GAS strains revealed 99% similarity with only 5 variable amino acids (Figure S1A). Due to the high amino acid conservation of SPy_0802 among GAS strains and the presence of homologous proteins limited to other *Streptococcus* species, we named this translational product S protein and its genetic locus *ess*.

### S Protein Is an Extracellular and Cell-Wall-Associated Protein

S protein was identified in our original work as a secreted protein (Lapek et al., 2017a), although it was detected in a previously published screen for membrane-bound GAS antigens (Rodríguez-Ortega et al., 2006). To addr*ess* these divergent findings, a recombinant version of the protein was purified (Figure S1B) and used to raise polyclonal rabbit antisera. Immunoblotting analyses indicated that S protein is abundant both as a cell-associated protein and in the extracellular milieu throughout GAS growth. In the supernatant, it undergoes proteolytic cleavage during stationary phase (Figures 1D and 1E) through a yet-to-be-determined mechanism.

### S Protein Governs Hydrophobic Properties of GAS Cells

To study the effect of S protein on GAS physiology, an allelic exchange deletion strain and a complemented deletion strain expressing S protein *in trans* (Δ*ess* pD*Cerm*::*ess*) were constructed in an M1 5448 background. We observed that the absence of S protein resulted in a striking difference in cell sedimentation (Figure 1F). We hypothesized that this distorted phenotype was related to a difference in cell morphology. We tested this using fluorescence microscopy of GAS cells. However, no difference in Δ*ess* cell chain length or diameter was detected (Figures S1C and S1D). We next sought to determine whether the sedimentation defect could be caused by differential proliferation rates caused by the absence of S protein. Measured viable cell counts indicated no difference in the Δ*ess* growth pattern or generation time (G) (Figure 1G). Analysis of GAS growth curves based on the optical density indicated that bacteria deprived of S protein are more dispersed in the medium (Figure 1H).

Upon further examination of GAS during growth, it was observed that at a certain density, cells expressing S protein form macro-structures (Figure 1I). Over time, these structures precipitate in culture. Furthermore, upon disruption, these macro-structures re-formed over time (Figure S1E).

Based on the different strain behavior in the culture medium, we hypothesized that formation and sedimentation of the cellular macro-structures are driven by the hydrophobic properties of the GAS cell surface. To test this, we analyzed GAS sedimentation in media with different chemical properties. WT and complemented strains sedimented in the water control medium and remained in solution upon addition of the water-miscible solvent methanol (Figures 1J and 1K). In contrast, Δ*ess* displayed an opposite behavior under these test conditions. Finally, we measured the GAS surface hydrophobicity based on the ability of cells to bind *n*-hexadecane (Rosenberg et al., 1980). Our data show that Δ*ess* binds l*ess n*-hexadecane than WT and complemented strains (Figure 1L). Spontaneous cell lysis was not observed for the above experiments (Figures S1F and S1G).

### Δ*ess* Is More Susceptible to Phagocytic Killing *In vitro*

**Figure 2.**
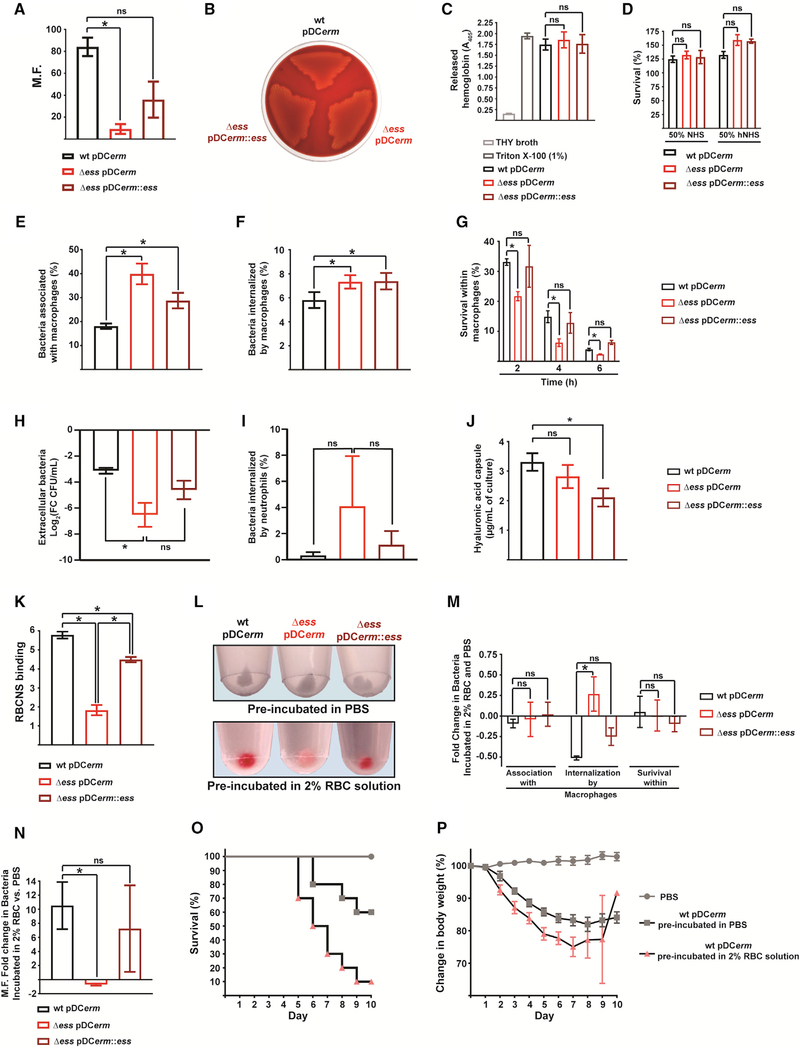
S Protein Is Crucial for Survival in Human Blood and Coating of GAS Cells with Lysed RBC Fragments (A) Proliferation of GAS strains in whole human blood, in biological triplicate with data as mean ± SEM (*p < 0.05). (B) Photographic documentation of hemolytic properties of GAS strains on 5% sheep blood agar. (C) Quantification of red blood cells lysis by GAS strains, in biological triplicate with data as mean ± SEM (*p < 0.05). (D) Bacterial survival in normal human serum (NHS), in biological triplicate with data as mean ± SEM (*p < 0.05). (E) Quantification of GAS cells captured by THP-1-derived macrophages, in biological triplicate with data as mean ± SEM (*p < 0.05). (F) Quantification of GAS cells phagocytosed by THP-1-derived macrophages, in biological triplicate with data as mean ± SEM (*p < 0.05). (G) Quantification of bacterial survival within THP-1-derived macrophages, in biological triplicate with data as mean ± SEM (*p < 0.05). (H) Quantification of recovered extracellular bacteria following incubation with neutrophils, in biological triplicate with data as mean ± SEM (*p < 0.05). (I) Quantification of bacterial cells phagocytosed by neutrophils, in biological triplicate with data as mean ± SEM (*p < 0.05). (J) Quantification of bacterial hyaluronic acid capsule, in biological triplicate with data as mean ± SEM (*p < 0.05). (K) Quantification of RBCNS binding by Δ*ess*, in biological triplicate with data as mean ± SEM (*p < 0.05). (L) Photographic documentation of pelleted GAS cell color after incubation in PBS or 2% RBC solution. (M) Effect of RBC membrane binding on interaction with THP-1 derived macrophages, in biological triplicate with data as mean ± SEM (*p < 0.05). (N) Effect of RBC membrane binding on proliferation of GAS strains in whole human blood, in biological triplicate with data as mean ± SEM (*p < 0.05). (O) Survival of mice (n = 10) infected with WT GAS preincubated in PBS or 2% mouse RBC solution. (P) Change in body weight of mice (n = 10) infected with WT GAS preincubated in PBS or 2% mouse RBC solution.

Blood survival and dissemination is a major virulence property of GAS and is achieved through several mechanisms (Fischetti, 2016). Because S protein was identified as an RBC-specific protein, we analyzed its importance for GAS growth in blood. We observed that Δ*ess* had a significantly decreased ability to grow in human blood compared to the WT and complemented strains (Figure 2A). Next, we analyzed the interaction of Δ*ess* with individual blood components. S protein was not required for β-hemolysis (Figures 2B and 2C). Because S protein is predicted to be peptidoglycan associated, we next endeavored to test whether S protein might act as a protective agent against complement killing. The absence of S proteins did not affect GAS resistance to complement-mediated killing (Figures 2D and S2A). Further probing of the blood components revealed that a lack of S protein results in GAS being more readily captured, phagocytized, and killed by THP-1-derived macrophages (Figures 2E–2G). We next assessed bacterial viability in the presence of human neutrophils. We found that Δ*ess* was more susceptible to extracellular killing in the presence of neutrophils. In our study of bacterial internalization by neutrophils, there was a trend toward increased uptake of Δ*ess* compared to WT and complemented strains (Figures 2H and 2I). Increased association and phagocytosis of Δ*ess* in the phagocytic cells was only marginally rescued in the complement strain. Therefore, we subsequently quantified the hyaluronic acid capsule, an anti-phagocytic GAS determinant (Wessels et al., 1991). This assay showed decreased encapsulation of the complemented strain but not of Δ*ess* (Figure 2J). These data support the notion that all phagocyte-related phenotypes of Δ*ess* are caused by a lack of S protein expression, while the elevated amount of complemented strain captured and internalized by phagocytes is a consequence of decreased capsule formation.

### Binding of RBC Membranes by Surface-Associated S Protein Facilitates GAS Virulence

Because a previous study showed that S protein present in bacterial culture supernatants selectively binds to RBC nanosponges (RBCNSs) (Lapek et al., 2017a), we investigated the interaction between RBCs and cell-associated S protein. To this end, we tested binding of RBCNSs by whole GAS cells. Corroborating previous findings, Δ*ess* bound significantly l*ess* RBCNSs than WT and complemented strains (Figure 2K).

Based on these observations, we hypothesized that S protein coopts host RBC membranes for immune camouflage. To test this, we evaluated interactions with macrophages of GAS preincubated in RBC solution or PBS. Upon 100% endogenous lysis of RBCs (Figure S2B), WT and complemented strains gained a prominent red color, whereas Δ*ess* became faintly pink, indicating reduced RBC binding in the absence of S protein (Figure 2L). RBC coating did not affect GAS viability or the amount of bacteria associating with or surviving within macrophages (Figures S2C and 2M). However, pre-incubation with RBCs decreased the phagocytic uptake of the WT and complemented strains, while increasing the uptake of Δ*ess*.

We next tested the effect of RBC coating on GAS survival in whole human blood. Supporting our *in vitro* macrophage data, pre-incubation with RBCs increased the proliferation rate of the WT and complemented strains and decreased the viability of Δ*ess* when compared to bacteria preincubated with PBS (Figure 2N). Finally, we analyzed the effect of RBC coating on GAS virulence *in vivo* by using a mouse model of systemic infection (Lapek et al., 2018). In the tested cohort, mortality rates of 40% and 90% were observed for mice infected with uncoated and RBC-coated WT GAS, respectively (Figure 2O). Furthermore, the majority of the animals infected with GAS preincubated with RBCs displayed a more rapid decrease in body weight (Figure2P;TableS1).

### Absence of S Protein Reshapes GAS Cellular and Extracellular Proteomes

**Figure 3.**
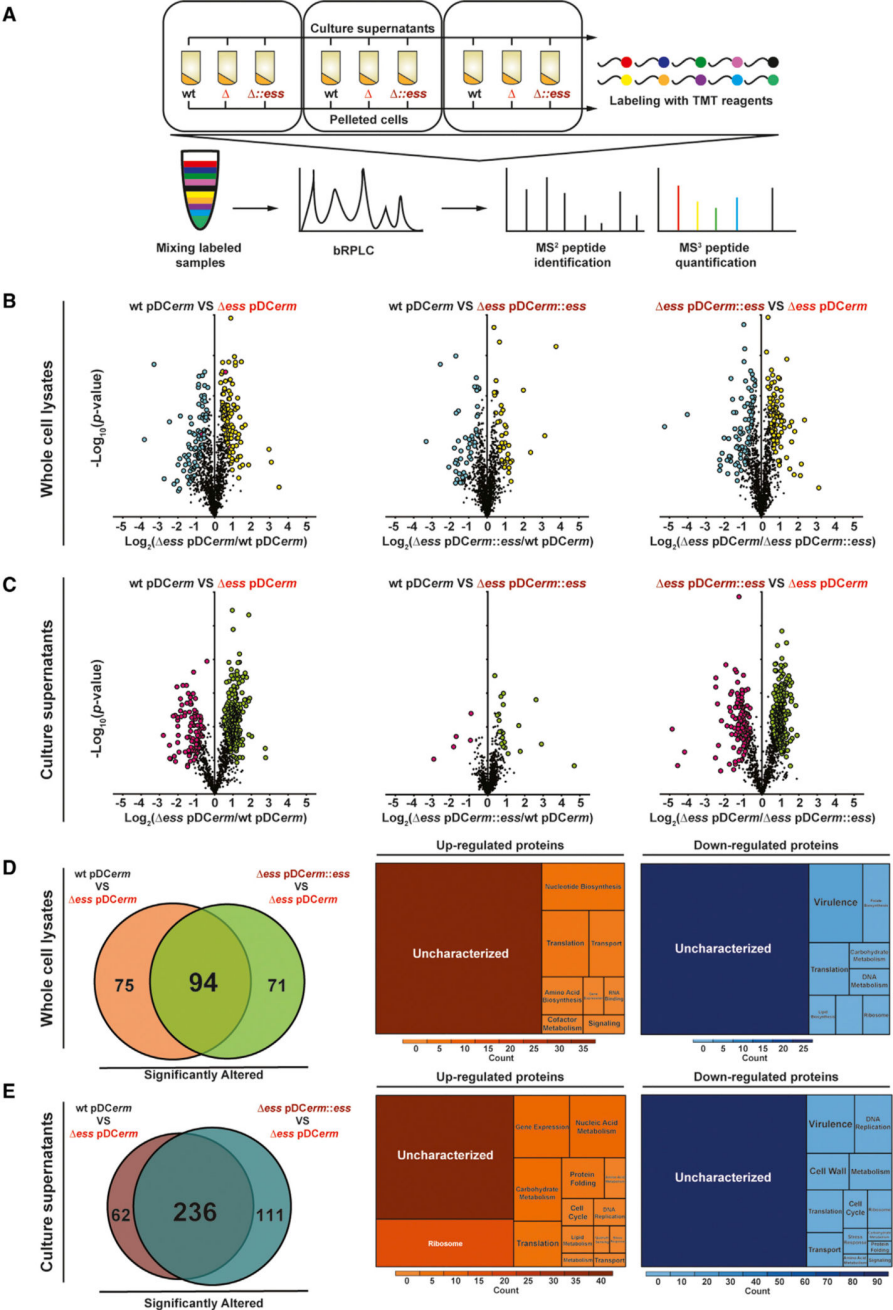
Absence of S Protein Reshapes Cellular and Extracellular Proteome Landscape (A) Outline of proteomic workflow for GAS cells and supernatants collected as biological triplicates. (B) Binary comparisons of protein abundance among analyzed bacterial strain cells. Proteins with π > 1.1082 are highlighted in cyan and yellow. (C) Binary comparisons of protein abundance among analyzed bacterial strain supernatants. Proteins with π > 1.1082 are highlighted in pink and green. (D) Core set of GAS whole-cell lysate proteome components affected by S protein. Orange and blue treemaps represent distribution of up- and downregulated proteins, respectively, among functional groups. (E) Core set of GAS culture supernatant proteome components affected by S protein. Orange and blue treemaps represent distribution of up- and downregulated proteins, respectively, among functional groups.

To gain a more comprehensive understanding of the role S protein plays in GAS physiology, we performed a quantitative proteomic analysis of WT, Δ*ess*, and complemented strain whole-cell lysates and culture supernatants (Figure 3A). Whole-cell lysate analysis revealed several differentially abundant proteins as follows: 203 between WT and Δ*ess*, 90 between WT and complemented, and 185 between complemented and Δ*ess* (Figure 3B; Table S2). Results from the culture supernatant proteome analysis were even more striking, with numbers of proteins showing altered expression as follows: 305 between WT and Δ*ess*, 19 between wild and complemented, and 352 between complemented and Δ*ess* (Figure 3C; Table S2). We excluded from further analysis proteins that were either similarly dysregulated in Δ*ess* and complemented strains in comparison to the WTstrain or exclusively altered in the complemented strain (with exception to S protein). Through the comparison of Δ*ess* to the WT and complemented strain, we defined a core set of 94 and 236 cellular and extracellular proteome components affected by S protein, respectively (Figures 3D and 3E). The vast remodeling of the proteome landscape in Δ*ess* is may be at least in part driven by the change in expression of four putative transcription factors, although this hypothesis requires further investigation.

Classification of S-protein-dependent proteins revealed that they belong to multiple functional categories, although most were uncharacterized (Figures 3D and 3E). Notably, in the absence of S protein, many recognized virulence factors critical for invasive disease were downregulated in bacterial cells and culture supernatants (Figures S3A and S3B).

Among the virulence factors downregulated in Δ*ess* was M protein, a widely studied and highly abundant GAS virulence factor (Fischetti, 2016). Several studies have demonstrated the role of M protein in GAS surface hydrophobicity and bacterial aggregation (Frick et al., 2000; Tylewska et al., 1979). Because our data show that Δ*ess* displays defects in cell sedimentation and surface hydrophobicity (Figures 1F and 1I–1L), we investigated whether these phenotypes were related to downregulation of M protein or whether they were S protein specific. We introduced into Δ*ess* a vector containing the *emm1* gene under the control of a constitutive promoter (pDC*erm*::*emm1*) to elevate M protein abundance (Figure S3C).

The Δ*ess* pDC*erm*::*emm1* showed significantly increased amounts of sedimented cells compared to Δ*ess*, although this strain did not rescue sedimentation to the level of Δ*ess* pDC*erm*::*ess*. This suggests that both S and M proteins play a role in sedimentation (Figure S3D). Similarly, elevating M protein abundance in Δ*ess* drastically decreased the amount of non-sedimented cells remaining in solution (Figure S3E). Because no difference in viability was observed among the tested strains (Figure S3F), we concluded that the measurable change in cell aggregation is related to M-protein-mediated cell-cell interactions rather than differences in bacterial abundance or stage of growth. Subsequent studies showed that increased abundance of M protein did not affect *n*-hexadecane binding by Δ*ess* (Figure S3G). We conclude that downregulation of M protein affects Δ*ess* cell aggregation but not surface hydrophobicity.

Given the large-scale proteome reorganization in the Δ*ess* strain, we next investigated the hypothesis that S protein bears direct responsibility for binding RBC membranes. To test this, we used serum harvested from S-protein-immunized rabbits to pre-block WT GAS cells. Compared to bacteria blocked with serum collected from naive rabbits, GAS preincubated in anti-S protein serum bound l*ess* RBC membranes (Figures S3H and S3I). Endogenous hemolysis and recovered bacterial CFUs were equivalent between bacteria preincubated in naive and anti-S protein sera (Figures S3J and S3K). Based on these data, we conclude that S protein bears a degree of direct responsibility for binding RBC membranes.

### Δ*ess* Mutant Has Highly Attenuated Virulence in a Mouse Model of Systemic Infection

**Figure 4.**
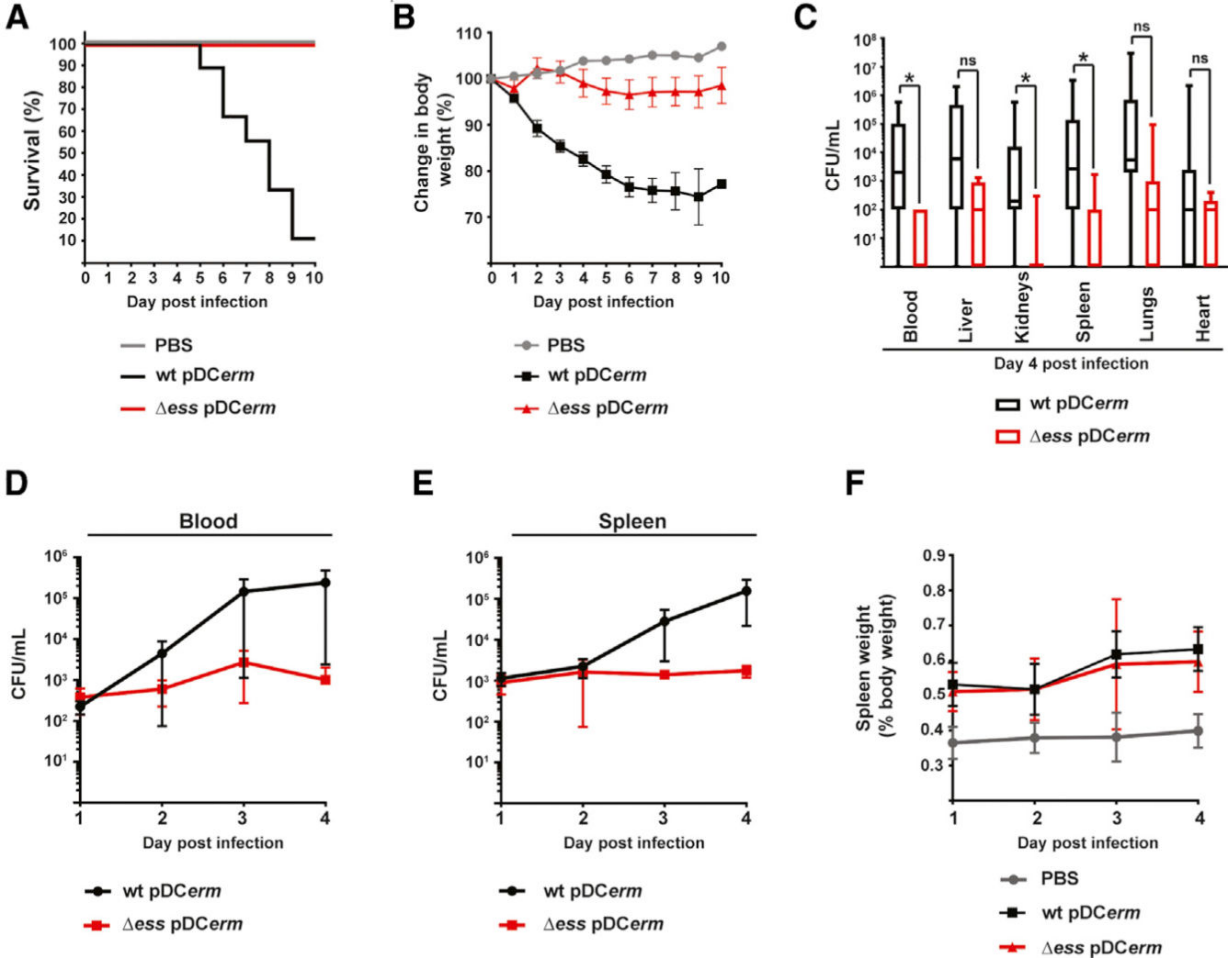
Δ*ess* Shows Attenuated Virulence in a Mouse Model of Systemic Infection (A) Survival of mice (n = 10) infected with GAS WT *and* Δ*ess* strains. (B) Change in body weight of mice (n = 10) infected with GAS WT and Δ*ess* strains, as mean ± SEM. (C) Bacterial loads in mouse blood and organs on day 4 of systemic infection (n = 7), as mean ± SEM (*p < 0.05). (D) Progression of bacterial burden in the blood during 4 initial days post-infection (n = 5), as mean ± SEM. (E) Progression of bacterial burden in splenic tissues over 4 days post-infection (n = 5), as mean ± SEM. (F) Changes in the size of spleens during infection (n = 5), as mean ± SEM.

Because Δ*ess* displayed a clear decrease in virulence *in vitro*, we hypothesized that S protein plays an important role during infection *in vivo*. To test this, we used a mouse model of systemic infection (Lapek et al., 2018; Lin et al., 2015). During a 10-day trial, WT GAS was associated with a progressive decrease in animal body weight and 90% mortality rate (Figures 4A and 4B). In contrast, all mice infected with Δ*ess* survived the challenge and their body weight stabilized and remained constant after a slight initial decline. Dissection of animals at day 4 post-infection revealed that Δ*ess* was largely cleared from the bloodstream (Figure 4C). There was also an overall lower bacterial load in their organs compared to WT GAS. The S-protein-complemented strain was also included in the *in vivo* experiments. However, due to poor maintenance of pDC*erm*::*ess* vector, no significant difference between the complemented strain and Δ*ess* was observed *in vivo* (Figures S4A–S4D).

Subsequent analyses of GAS load throughout the initial days of infection revealed that 24 h following infection there were approximately equivalent levels of the WT and Δ*ess* GAS detected in the blood and splenic tissues (Figures 4D and 4E). Although levels of WT GAS in the blood increased almost logarithmically over the course of the subsequent 3 days, the amount of Δ*ess* increased only slightly by day 3 and began to decline at day 4. Bacterial load of WT GAS in the spleens also increased over time, whereas levels of Δ*ess* remained similar throughout the infection. We also observed that during infection, the spleens of animals infected with WT and Δ*ess* were significantly enlarged in comparison to control animals (Figure 4F). To ensure that the striking *in vivo* attenuation phenotype observed in Δ*ess* was not associated with any unexpected mutations garnered during the generation of this strain, whole-genome sequencing was performed. This analysis did not reveal any mutations in *emm1* or other known virulence factors downregulated in the Δ*ess* proteome data, including known master regulators of GAS virulence (Churchward et al., 2009; Graham et al., 2002; Manetti et al., 2007; Metzgar and Zampolli, 2011; Sumitomo et al., 2013).

### S Protein Deficiency Leads to Elevated Immune Pathway Signaling and Is Associated with Robust Learned Immunity

**Figure 5.**
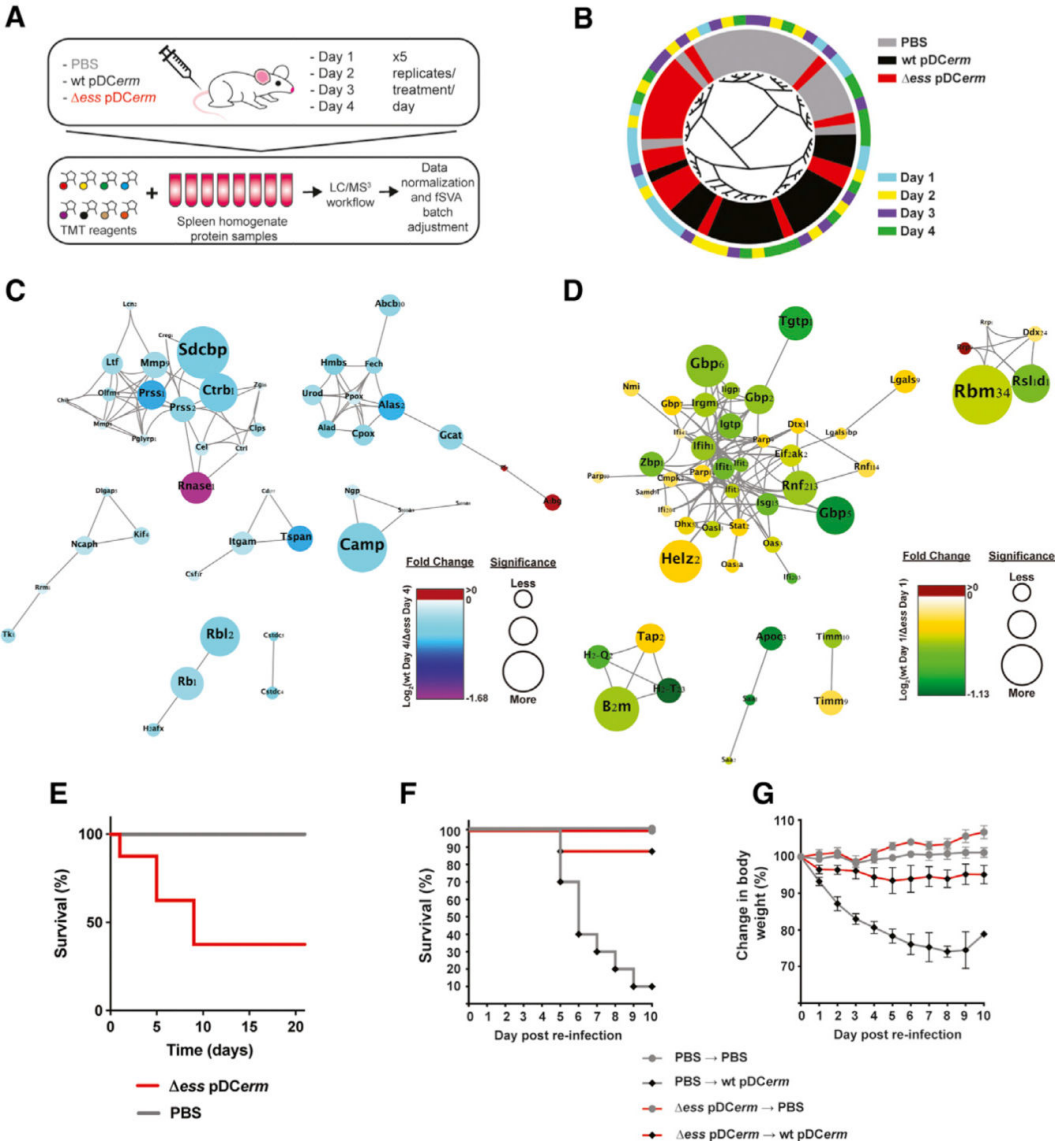
Lack of S Protein Leads to Elevated Interferon-Related Signaling during Early Infection and Adaptive Immunity Development (A) Outline of the quantitative proteomic workflow for splenic tissues harvested from PBS mock-infected and GAS-infected mice on days 1–4 (n = 5). (B) Circular dendrogram of Spearman clustering for all samples. Treatment and time point are represented in the inner and outer circle, respectively. (C) Protein interaction network of Δ*ess*-infected spleen cluster 7 using day 4 abundance values. (D) Protein interaction network of Δ*ess* infected spleen cluster 0 and 1 using day 1 abundance values. (E) Survival of Ifnar1^−/−^ mice following Δ*ess* infection (n = 8). (F) Survival of PBS- or Δ*ess*-inoculated mice during re-infection with WT GAS (groups: PBS → PBS, n = 10; PBS → WT, n = 10; Δ*ess* → PBS, n = 8; Δ*ess* → WT, n = 8). (G) Change in body weight of PBS- or Δ*ess*-inoculated mice during re-infection with WT GAS (groups: PBS → PBS, n = 10; PBS → WT, n = 10; Δ*ess* → PBS, n = 8; Δ*ess* → WT, n = 8). Data are represented as mean ± SEM.

The spleen is an important secondary lymphoid organ responsible for coordinating innate and adaptive immune responses against bacterial pathogens filtered from the blood (Bronte and Pittet, 2013). Based on the dynamics of WT and Δ*ess* bacterial burden in the blood and spleens over the course of infection, we hypothesized that the absence of S protein alters the host immune response. To better understand differential immune responses associated with infection with the WT and mutant strain, we performed a tandem mass tag (TMT)-based temporal proteomic analysis of spleens harvested from WT, Δ*ess*, or PBS mock-infected mice on days 1 through 4 post-infection (Figure 5A; Table S3). Upon batch adjustment, the proteome data clustered largely by treatment group (Figure 5B). Short Time-series Expression Miner (STEM) clustering was used to identify temporal trends in spleen proteomics data (Figure S5A; Table S4) (Ernst and Bar-Joseph, 2006).

STEM clustering of Δ*ess* spleen proteome data demonstrated one group of proteins that appeared to increase in abundance over time (cluster 7). The upward trend of these proteins appeared to be largely absent during WT or mock infection (Tables S3 and S4). We next subjected the proteins within these clusters to interaction network analysis using String, focusing on protein abundance differences between WT and Δ*ess* infection cohorts at day 1. String analysis revealed a cluster of related proteins involved in antimicrobial activity or immune modulation (Figure 5C).

In the Δ*ess* infection clusters, we also identified two groups of proteins that began at high levels on day 1 and showed a sustained decrease in abundance over time (clusters 0 and 1). We found that this protein abundance trend was unique to Δ*ess* and that mice infected with WT GAS or administered PBS did not show striking changes in these proteins over the infection time course, including at day 1 (Tables S3 and S4). As expected, String analysis revealed multiple immune-related protein clusters. The most prominent included several type I interferon (IFN)-response proteins. Many of these proteins followed the temporal trend seen in clusters 0 and 1, spiking on day 1 of Δ*ess* infection, and then dropping to levels found in the mock-infected animals (Figure S5C). Notably, bacterial burden in blood and spleens at day 1 post-infection was roughly equivalent between WT and Δ*ess*-infected animals (Figures 4D and 4E). The observed pattern of IFN signaling closely matched clearance of Δ*ess* during infection (Figure 4D). These results suggested that during exposure to lethal levels of GAS, IFN signaling is partially responsible for the positive outcomes associated with infection with Δ*ess*. To test whether type I IFN signaling was responsible for enhanced survival of Δ*ess*-infected mice, we infected Ifnar1^−/−^ mice with Δ*ess* and monitored survival for 3 weeks. Whereas earlier studies demonstrated 100% survival of mice infected with Δ*ess* (Figure 4B), only 38% of Ifnar1^−/−^ mice survived infection with Δ*ess* (Figure 5E).

Because we saw robust engagement of core immune pathways during the early stages of infection with Δ*ess*, we hypothesized that infection with a strain lacking S protein might elicit long-term immune memory. To test this, we exposed mice to systemic infection with Δ*ess* or PBS. After 3 weeks, equal numbers of mice from each group were administered PBS or infected with WT GAS. Mice initially injected with PBS and later infected with WT GAS displayed high mortality (90%) and a decrease in body weight throughout infection (Figures 5F and 5G). In contrast, 7 out of 8 mice initially exposed to Δ*ess* survived the challenge with the WT strain and showed no progressive loss in body weight. Collectively, we show that infection with Δ*ess* stimulates IFN signaling and that challenge with this strain results in robust protection against GAS infection.

## DISCUSSION

Despite decades of research and rapid technological advancements, our understanding of bacterial physiology and host-pathogen interactions remains limited. Protein products of approximately 50% of bacterial genes are either hypothetical or of unknown function (Sivashankari and Shanmughavel, 2006). Understanding how these uncharacterized proteins affect bacterial pathogenicity is critical for the formulation of alternative pharmacological interventions, which is increasingly important in the era of wide-spread antibiotic resistance (Zaman et al., 2017). In this work, we have performed an initial characterization of S protein, a previously uncharacterized protein, and investigated its involvement in GAS pathogenesis.

Pathogens apply a multitude of mechanisms to avoid recognition by the host immune system. Imitation of host structures, known as the molecular mimicry, is one such strategy (Wessels et al., 1991). Herein, we described a form of molecular mimicry where GAS uses S protein to bind RBC fragments. Sequestration of RBC fragments prevents phagocytosis of GAS by macrophages and amplifies virulence *in vitro* and *in vivo* (Figures 2M–2P). GAS hemolysis has been intensively studied. However, the evolutionary rationale behind this proc*ess* remains incompletely understood. The discovery that GAS utilizes lysed RBCs as immune camouflage provides a link between hemolysis, a hallmark diagnostic phenotype of GAS in the clinical laboratory, and an infectious process of the pathogen.

Proteomic profiling of Δ*ess* indicated that the absence of S protein results in a vast rearrangement of the GAS cellular and extracellular proteome. These results suggest that S protein is a multifunctional protein that, besides directly participating in virulence, could be additionally involved in gene expression regulation or in maintaining proteome homeostasis. Protein moonlighting, wherein a single protein performs more than one function, is well recognized in the literature (Jeffery, 1999, 2003, 2014). Among proteins downregulated in Δ*ess* were several known virulence factors, including M protein (Table S2; Figures S3A and S3B). M protein is a crucial virulence determinant of GAS required for survival in whole human blood and avoidance of phagocytosis (Fischetti, 2016). Diminished levels of M protein could explain increased susceptibility to *in vitro* phagocytic killing of the Δ*ess* strain in the absence of RBC coating (Figures 2E and 2I).

We observed that during early Δ*ess* bacteremia, rapid disease clearance was associated with a distinct spike in IFN-regulated proteins early in infection (Figures 4D, 4E, and 5D). We showed that IFN signaling was necessary for Δ*ess* bacterial clearance (Figure 5E). Subsequently, it was found that Δ*ess* infection was associated with protection from reinfection with WT GAS (Figures 5F and 5G). It was previously shown that GAS stimulates production of type I and II IFN in human and murine cells and that Ifnar^−/−^ mice more readily succumb to GAS infection than WT mice (Cavaillon et al., 1982; Goldmann et al., 2007; Gratz et al., 2011; Hyland et al., 2009; Miettinen et al., 2000; Müller-Alouf et al., 1997). In this study, WT GAS infection was not associated with a spike in IFN levels on days 1–4 post-infection. Although inhibition of IFN signaling was not investigated as a GAS virulence strategy, our results suggest that robust GAS infection is associated with low viability of this immune pathway.

Together, the findings regarding S protein reported in this work can have a 2-fold impact on the development of countermeasures against GAS. First, due to its highly conserved nature among GAS serotypes and involvement in virulence, S protein is an ideal target for anti-virulence therapeutics. Inactivation of S protein function would make GAS vulnerable to the host immunity. Second, we identified immune pathways that are strongly associated with positive outcomes against GAS infection. These host factors could be a starting point for future investigation into host-centered GAS therapies. Ultimately, our findings on S protein suggest that key presumptions regarding GAS infectious disease pathogenesis may be incomplete and require reevaluation.

## STAR★METHODS

### LEAD CONTACT AND MATERIALS AVAILABILITY

Further information and requests for resources and reagents including engineered bacterial strains should be directed to and will be fulfilled by the Lead Contact, David J. Gonzalez (djgonzalez@ucsd.edu). All unique/stable reagents generated in this study are available from the Lead Contact without restriction.

### EXPERIMENTAL MODEL AND SUBJECT DETAILS

#### Mice

ICR (CD-1) Outbred Mice were ordered from Envigo. *Ifnar1* −/− mice were ordered from the MMRRC. Animals were housed in a Specific Pathogen-Free facility, with 2–3 specimens per individually-ventilated cage with aspen chip bedding. All studies using CD-1 mice used female mice between 6–8 weeks old. *Ifnar1* −/− mice were 13 week old females ordered from Jackson Laboratories via MMRRC Jax and were housed as described above. All animal experiments were performed in accordance with NIH guidelines and approved by the Institutional Animal Care and Use Committee (IACUC) of UCSD under protocol S09388.

#### Bacteria

*Streptococcus pyogenes* M1 5448 (Chatellier et al., 2000) and its derivatives were cultured in Todd Hewitt Broth (Spectrum Laboratory Products, Inc.) supplemented with 0.2% yeast extract (VWR International, LLC) (THY) or grown on solid THY with addition of 1.4% agar (VWR International, LLC) or Mueller Hinton Agar with 5% Sheep Blood (Fisher Scientific) statically at 37°C, unless stated otherwise (Gera and McIver, 2013). Medium for *S. pyogenes* strains carrying pHY304-*ess*::*cat*, pDC*erm*, pDC*erm*::*ess*, or pDC*erm*::*ermm1* was supplemented with 2 μg/mL erythromycin (Spectrum Laboratory Products, Inc.). *Escherichia coli* strains NEB 5-alpha, NEB Turbo, and BL21(DE3) (New England BioLabs) and its derivatives were cultured in Luria-Bertani (LB) broth (Spectrum Laboratory Products, Inc.) at 37°C with aeration (220 rpm) or on LB agar (Core Bio Services) at 37°C, unless stated otherwise (Son and Taylor, 2012). The following antibiotics were used to supplement growth medium for *E. coli*: 250 μg/mL erythromycin for strains carrying pHY304, pHY304-*ess*::*cat*, pDC*erm*, pDC*erm*::*ess*, or pDC*erm*::*ermm1*; 50 μg/mL kanamycin (BioPioneer) for strains containing N-HisPP-pET-28a(+) or N-HisPP-pET-28a(+)::*ess*. Engineered *E. coli* and *S. pyogenes* strains were stored by mixing 700 μL of the overnight cultures with 300 μL of 50% sterile glycerol (VWR International, LLC) (final glycerol concentration 15%) or 600 μL of the overnight cultures with 400 μL of 50% sterile glycerol (final glycerol concentration 20%) respectively. Bacterial stocks were stored at −80°C.

#### Cell Lines

The acute monocytic leukemia THP-1 cells were acquired from American Type Culture Collection (ATCC). All cells were cultured in the RPMI 1640 Medium (ATCC modification) (Life Technologies Corporation) supplemented with 10% Fetal Bovine Serum (USDA Certified, Heat Inactivated) (Core Bio Services) and 0.05 mM 2-mercaptoethanol (Life Technologies Corporation) at 37°C in the presence of 5% CO_2_. Cells were maintained at density 4 ^×^ 10^5^ − 1 ^×^ 10^6^/mL. Differentiation of THP-1 cells into macrophages was achieved by supplementing the medium with 25 nM phorbol 12-myristate 13-acetate (PMA) (Sigma Aldrich) and is described in the Method Details section.

### METHOD DETAILS

#### Genetic Manipulations of Streptococcus pyogenes (GAS) M1 5448

Wizard Genomic DNA Purification Kit (VWR International, LLC) was used for isolation of bacterial genomic DNA. Q5 High-Fidelity DNA Polymerase (New England BioLabs) was used for amplification of DNA designated for molecular cloning or sequencing purposes. QIAGEN Plasmid Mini Kit (QIAGEN, Inc.) and QIAquick PCR Purification Kit (QIAGEN, Inc.) were used for purification of plasmid DNA and PCR products, respectively. Quick-Load® Taq 2X Master Mix (New England BioLabs) was used for analyses of *E. coli* and GAS clones. DNA sequencing was performed at the Retrogen Inc. core. The *ess* (*SPy_0802*) gene replacement by chloramphenicol resistance cassette (*cat*) by homologous recombination was performed based on a previously described protocol (Le Breton and McIver, 2013) with minor changes to preparation of electrocompetent GAS cells (Dunny et al., 1991). Primers for amplification and Gibson Assembly of the chloramphenicol resistance cassette (*cat*) flanked by up- and downstream regions of *ess* (*SPy_0802*) gene were designed using online NEBuilder Assembly Tool (http://nebuilder.neb.com/). Chromosomal DNA of M1 5448 was isolated and served as a template for PCR amplification of 500 base pair regions directly up- and downstream of the *ess* locus using primer pairs *ess*-up-F/*ess*-up-R and *ess*-down-F/*ess*-down-R. The *cat* gene was amplified using pACYC184 (Nakano et al., 1995) as a DNA template and primers *cat*-F and *cat*-R. PCR products were purified and assembled with NEBuilder HiFi DNA Assembly Master Mix (New England BioLabs). The assembly product was used as a template for amplification of the entire DNA region with primer pair *ess-cat*-F/*ess-cat*-R. Plasmid pHY304 (Pritzlaff et al., 2001) was PCR amplified using primers pHY304-F and pPHY304-R. PCR products were purified, subjected to digestion with EagI-HF and HindIII-HF restriction enzymes (New England BioLabs), purified, and ligated using Quick Ligation Kit (New England BioLabs). The entire ligation product was used for transformation of *E. coli* NEB Turbo competent cells (New England BioLabs), according to the manufacturer’s recommendation. As pHY304 contains a thermosensitive origin of replication that replicates at 30°C, the outgrowth of bacteria in SOC medium (New England BioLabs) and overnight incubation on solid medium supplemented with erythromycin was performed at 30°C. Bacterial colonies were passaged onto fresh solid medium and screened for the presence of pHY304-*ess*::*cat* by PCR reaction with primers pHY304-Ver-F and pHY304-Ver-R (each primer is complementary to the vector region approximately 200 base pairs up- and downstream respectively from the insertion site. The DNA template for the PCR reaction was obtained by suspending minute amounts of bacterial cells in 30 μL of sterile ddH_2_O and incubating for 5 minutes at 100°C). Plasmid DNA form positively identified bacterial clones was purified and verified by DNA sequencing with primers pHY304-Ver-F and pHY304-Ver-R.

Electrocompetent GAS cells were prepared as follows: overnight cultures of M1 5448 supplemented with 20 mM glycine (Spectrum Laboratory Products, Inc.) were back-diluted (1:20) in 150 mL of THY + 20 mM glycine, incubated until they reached OD_600_ = 0.2–0.4, and cooled on ice for 30 minutes. The culture was spun down for 20 minutes at 10,000 ^×^ *g*, 4°C, and cells were washed three times with ice-cold 0.625 M Sucrose (Sigma Aldrich), 1 mM MgCl_2_ ⋅ 6H_2_O (Fisher Scientific). Cells were finally suspended in 1 mL of 0.625 M Sucrose, 1 mM MgCl_2_ ⋅ 6H_2_O and 50 μL aliquots were used immediately or stored at −80°C. M1 5448 cells were transformed with 10 μg of pHY304-*ess*::*cat* by electroporation using BioRad Gene Pulser II and Gene Pulser/MicroPulser Electroporation Cuvettes, 0.2 cm gap (BioRad) (cooled on ice before application) at the following settings: voltage 1.75 kV, resistance 400 Ω; capacitance: 25 μF. Electroporated cells were mixed with 500 μL of THY broth and 0.25 M Sucrose, and were transferred to an Eppendorf tube. They were incubated at 30°C for 2 hours and plated on solid medium supplemented with erythromycin. Following 2 days of incubation at 30°C, bacterial colonies were passaged onto fresh agar plates with antibiotic and verified for the presence of pHY304-*ess*-*cat* as described above for *E. coli*. M1 5448 pHY304-*ess*::*cat* was cultured overnight at 30°C in 10 mL of THY broth supplemented with erythromycin, serially diluted in PBS (Cell Signaling Technology, Inc.), plated (dilutions 10^−3^ − 10^−5^) onto solid medium containing erythromycin, and incubated overnight at 37°C. On the following day, bacterial colonies with vectors that had integrated into the chromosome were inoculated in 10 mL of THY broth supplemented with erythromycin, and cultured overnight at 37°C. Frozen stocks were prepared as described above. On the following day, bacteria with an integrated pHY304-*ess*::*cat* plasmid (indicating a single crossover event) were plated onto solid medium without antibiotic supplementation and incubated overnight at 30°C. An individual bacterial colony was next inoculated in antibiotic-free medium and incubated at 30°C. On the following day, the culture was back-diluted (1:1000) in 10 mL of medium without antibiotic and incubated overnight at 37°C. The back-dilution and overnight incubation at 37°C was repeated. The culture was subsequently serially diluted in PBS and plated (dilutions 10^−3^ − 10^−5^) onto solid medium without erythromycin. Bacterial colonies were next passaged in parallel onto medium with and without erythromycin. Bacterial clones that that did not grow on medium supplemented with erythromycin (indicating a double crossover event) were verified by PCR (DNA extracted by boiling in water as described above) with the primer pair *ess*Del-Ver-F/*ess*Del-Ver-R. Primers are complementary to the chromosomal region approximately 700 base pairs from the beginning and the end of *ess* gene, and 200 base pairs up- and downstream of homologous recombination sites, respectively. Positively identified Δ*ess* clones were further verified by DNA sequencing of a PCR-amplified 700 base pairs up- and downstream region from the allelic exchange site (*ess*Del-Ver-F/*ess*Del-Ver-R primers were used for PCR amplification and sequencing).

The deletion of *ess* was genetically complemented through expression of *ess in trans* from a pDC*erm* vector (Jeng et al., 2003). The coding sequence of *ess* with a 319 base pairs upstream region containing predicted transcriptional promoter region(s) by BPROM (http://www.softberry.com/berry.phtml?topic=bprom&group=programs&subgroup=gfindb) and Promoter Prediction by Neural Network (http://www.fruitfly.org/seq_tools/promoter.html), and 100 base pairs downstream containing a transcriptional termination site were amplified with primer pair *ess*-F/*ess*-R. The 3335 base pairs of pDC*erm* was amplified with primers pDC*erm*-F and pDC*erm*-R. Both PCR products were purified, treated with KpnI-HF restriction enzyme (New England BioLabs), re-purified, ligated as described above, and the entire reaction product was used to transform of *E. coli* NEB 5-alpha competent cells (New England BioLabs), according to the manufacturer’s recommendation. Bacterial clones were passaged and screened for the presence of pDC*erm*::*ess* by PCR with primers pDC*erm*-Ver-F and pDC*erm*-Ver-R. Primer are complementary to the vector region approximately 200 base pairs up- and downstream form the insertion site, respectively. The DNA template for PCR was obtained as described above. Clones of pDC*erm*::*ess* were verified by DNA sequencing with primers pDC*erm*-Ver-F and pDC*erm*-Ver-R. Electrocompetent Δ*ess* cells were prepared and electroporated with 1 μg of either pDC*erm* or pDC*erm*::*ess* as described above. Cells were mixed with 500 μL of THY broth, 0.25 M Sucrose, transferred to an Eppendorf tube, incubated at 37°C for 1 hours, and plated on solid medium supplemented with erythromycin. Wild-type M1 5448 was simultaneously electroporated with pDC*erm*. GAS clones were verified for the presence of pDC*erm* vectors by PCR with the primer pair pDC*erm*-Ver-F/pDC*erm*-Ver-R as described above.

Genetic complementation of *ess* deletion with *emm1* expressed *in trans* from a pDC*erm* vector was performed in an analogous manner as described above. The DNA region encoding the M protein with 20 base pairs upstream including a native ribosome binding site and 100 base pairs downstream containing a transcriptional terminator were amplified with primers *emm1*-F and *emm1*-R. The PCR product was next digested with KpnI-HF enzyme, ligated into a PCR amplified pDC*erm* vector, and introduced into *E. coli* NEB Turbo competent cells. The verified pDCerm::*emm1* vector was next used for transformation of Δ*ess* cells. All primers used can be found in Table S5.

#### Purification of Recombinant S Protein and Production of Polyclonal Rabbit Antibodies

A recombinant version of S protein containing an N-terminal 6 × His-tag was obtained by amplification of the DNA region encoding S protein, excluding the start codon, with primer pair r*ess*-F/r*ess*-R. The purified 502 base pair PCR product and N-HisPP-pET-28a(+) (Buffalo et al., 2016) were cleaved with BamHI-HF and EagI-HF restriction enzymes (New England BioLabs), re-purified, ligated, and introduced into *E. coli* NEB 5-alpha competent cells as described above. Individual bacterial colonies were passaged onto fresh solid medium and screened for the presence of N-HisPP-pET-28a(+)::*ess* by PCR with the primers, NHpET28-Ver-F and NHpET28-Ver-R, binding approximately 200 base pairs up- and downstream respectively form the insertion site (preparation of the DNA template for PCR was performed as described above). The obtained vector, which was verified by DNA sequencing with primers NHpET28-Ver-F and NHpET28-Ver-R, was used for the transformation of *E. coli* BL21(DE3) competent cells (New England BioLabs), according to the manufacturer’s recommendation.

For the production of recombinant S protein, overnight cultures of *E. coli* BL21(DE3) N-HisPP-pET-28a(+)::*ess* was back-diluted (1:100) in 200 mL of LB supplemented with kanamycin and cultured to OD_600_ of approximately 0.5. Recombinant protein expression was induced through addition of isopropyl β-D-1-thiogalactopyranoside (IPTG) (Omega Scientific) to a final concentration of 0.05 mM, and the culture was incubated on a shaker overnight at 18°C and 220 rpm. Bacterial cells were harvested by 10 minute centrifugation at 6,000 ^×^ g at 4°C and stored at −80°C until used.

Affinity purification of recombinant S protein was performed as follows: pelleted *E. coli* BL21(DE3) N-HisPP-pET-28a(+)::*ess* cells were suspended in 20 mL of lysis buffer composed of 20 mM Tris-HCl pH 8.0 (Avantor Performance Materials), 500 mM NaCl (Sigma Aldrich), 10 mM imidazole (Sigma Aldrich), 50 mM L-Arginine (VWR International, LLC), 50 mM L-Glutamic Acid (Spectrum Laboratory Products, Inc.), and 0.5% Triton X-100 (Fisher Scientific). The lysis buffer was supplemented with a 1/4 of a cOmplete, Mini EDTA-free Protease Inhibitor Cocktail tablet (Roche Diagnostics) and lysozyme (BioPioneer) to a final concentration of 1 mg/mL. Samples were incubated on ice for 30 minutes followed by 3 cycles of sonication using a Q500 QSonica sonicator (Qsonica) equipped with 1.6 mm microtip at an amplitude of 35%. The sonication protocol was as follows: 10 s sonication; 10 s break; total sonication time 2 minutes; with 5 minutes incubation on ice in between cycles. Samples were subjected to centrifugation at 16,000 ^×^ *g* at 4°C to remove cell debris and unbroken cells. Supernatants containing soluble proteins were next passed through a 0.22 μm filter (MilliporeSigma) and incubated for 1 hour at 4°C with rotation with 1 mL of Ni-NTA agarose (QIAGEN) equilibrated with lysis buffer. Subsequently, the sample was loaded onto a Poly-Prep Chromatography Column (Bio-Rad Laboratories) and had been washed with 30 mL of wash buffer I (20 mM Tris-HCl pH 8.0, 500 mM NaCl, 40 mM imidazole, 50 mM L-Arginine, 50 mM L-Glutamic Acid, 0.5% Triton X-100) and 10 mL of wash buffer II (20 mM Tris-HCl pH 8.0, 500 mM NaCl, 40 mM imidazole, 50 mM L-Arginine, 50 mM L-Glutamic Acid, 0.05% Triton X-100). Recombinant S protein was eluted from the Ni-NTA agarose with 4 mL of elution buffer (20 mM Tris-HCl pH 8.0, 500 mM NaCl, 250 mM imidazole, 50 mM L-Arginine, 50 mM L-Glutamic Acid). In order to remove all Triton X-100, the sample was incubated for 2 hour rotating at room temperature with 1 g of Bio-Beads SM-2 Adsorbent Media (Bio-Rad Laboratories) that was initially equilibrated with elution buffer. Samples were separated from the adsorbent media by elution through a Poly-Prep Chromatography Column. Samples were transferred to dialysis tubing with molecular weight cutoff 6–8 kDa (Fisherbrand), and dialyzed against 10 mM Tris-HCl pH 8.0, 100 mM NaCl, 50 mM L-Arginine, 50 mM L-Glutamic Acid overnight at 4°C. The total concentration of recombinant S protein was measured using the DC Protein Assay (Bio-Rad Laboratories).

Rabbit polyclonal Anti-S protein antibodies against recombinant S protein were prepared by Pacific Immunology (https://www.pacificimmunology.com/) using a 13-week antibody production protocol and two New Zealand White rabbits (Animal Protocol #1 approved by IACUC and the NIH Animal Welfare Assurance Program No. A4182–01; US Department of Agriculture 93-R-283).

#### Growth in THY Broth

To determine growth rates of GAS strains, overnight cultures of M1 5448 wt pDC*erm*, Δ*ess* pDC*erm*, and Δ*ess* pDC*erm*::*ess* were back-diluted (1:20) in 10 mL of fresh medium and incubated for 7 hours at 37°C. Cultures were vortexed at each time point and optical densities at wavelength 600 nm (OD_600_) were measured with the use of SPECTRONIC 200 Spectrophotometer (Thermo Fisher Scientific). A sample of the culture was then serially diluted (10^−1^ − 10^−5^) in PBS and 5 μL spotted on solid medium for colony forming units (CFU) enumeration (calculated as CFU/mL). Experiments were performed in three biological replicates. Bacterial Generation Time (G) was calculated using the equation: G = (*t* /3:3 log *b* /*B*), where t = time interval (180 min), B = average number of bacteria at the beginning of the exponential phase of growth (2 hour time point), and b = the average number of bacteria at the end of the exponential phase of growth (5 hour time point). Bacterial cultures were documented by taking pictures with a Nikon D7000 digital camera equipped with macro lens.

#### Fluorescence Microscopy

To visualize the morphology of GAS cells, bacteria from late stationary (overnight cultures) or exponential (4 hours of growth post 1:20 back-dilution of overnight culture in fresh medium) phase of growth were mixed by vortexing. 6 μl of each cell suspension was added to 1.5 μl dye mix composed of 60 μg/mL FM4–64 (Life Technologies Corporation), 10 μg/mL DAPI (Sigma Aldrich), and 2.5 μM SYTOX Green (Life Technologies Corporation) in 1X T-base (2 g (NH_4_)_2_SO_4_ [Fisher Scientific], 18.3 g K_2_HPO_4_ ⋅ 3H_2_O [Fisher Scientific], 6 g KH_2_PO_4_ [Fisher Scientific], 1 g C_6_H_5_O_7_Na_3_ ⋅ 2H_2_O [Fisher Scientific] per 1 L of ddH_2_O) and transferred onto an agarose pad (20% LB broth, 1% agarose [Fisher Scientific]). Samples were air-dried under a fume hood (care was taken to prevent over-drying). Cells were visualized on an Applied Precision DV Elite optical sectioning microscope equipped with a Photometrics CoolSNAP-HQ2 camera. Pictures were deconvolved using SoftWoRx v5.5.1 (Applied Precision). Images for figures were prepared using FIJI. Bacterial cell diameters from multiple pictures were quantified with CellProfiler (Kamentsky et al., 2011) on two separate occasions.

#### Quantification of GAS Cells Sedimentation in THY Broth

Overnight cultures of M1 5448 wt pDC*erm*, Δ*ess* pDC*erm*, and Δ*ess* pDC*erm*::*ess* were mixed by vortexing. 3.6 mL were transferred to 4.5 mL disposable, polystyrene cuvette (Fisher Scientific), and mixed with either 400 μL of ddH_2_O or methanol (Fisher Scientific). Bacteria were mixed by pipetting and cuvettes were sealed with parafilm (Bemis Company Inc.). OD_600_ was measured from the side of the cuvette with SPECTRONIC 200 Spectrophotometer every 15 minutes for a total of 5 hours at room temperature. Bacterial cell sedimentation was measured as a “Change in OD_600_” which was calculated using equation: (*T* /*TO*)×100, where T = OD_600_ at the indicated time point, T0 = initial OD_600_ at time 0. In order to determine if spontaneous cell lysis had occurred during the experiment, bacteria were mixed by pipetting and OD_600_ values were recorded at the terminal time point. Experiments were performed in three biological replicates.

#### Binding of Bacterial Cells to n-hexadecane

Surface hydrophobicity of GAS cells measured by the ability to adhere to n-hexadecane was performed based on a previously described protocol (Ofek et al., 1983; Rosenberg et al., 1980). Overnight cultures of M1 5448 wt pDC*erm*, Δ*ess* pDC*erm*, and Δ*ess* pDC*erm*::*ess* were harvested by centrifugation for 10 minutes at 10,000 ^×^ *g* at room temperature, and pelleted cells were washed twice and suspended in PUM buffer (22.2 g K_2_HPO_4_ ⋅ 3H_2_0, 7.26 g KH_2_PO_4_, 1.8 g urea [Promega Corporation], 0.2 g of MgSO_4_ ⋅ 7H_2_0 [Sigma Aldrich], per 1 L of ddH_2_O). Next, 2.4 mL of the bacterial suspension was transferred into 13 ^×^ 100 mm borosilicate glass disposable culture tubes (Fisher Scientific) and 0.4 mL of *n*-hexadecane (Fisher Scientific) was added. Bacteria without addition of *n*-hexadecane were used as controls for spontaneous cell lysis. The OD_600_ was measured from the side of the tube using a SPECTRONIC 200 Spectrophotometer. Tubes were next vortexed for 3 minutes, allowed to settle for 15 minutes, and the OD_600_ of the bottom fraction was measured. Hydrophobic properties of bacterial cells are represented by the percentage of bacteria bound to the *n*-hexadecane, calculated using the following formula:((*T*0 *OD*600 − *T*15 *OD*600) /*T*0 *OD*600)×100, where T0 OD600 = OD_600_ value before vortexing and T15 OD600 = OD_600_ value after vortexing. Experiments were performed in three biological replicates.

#### Growth of GAS in Whole Human Blood

The ability of GAS strains to multiply in whole human blood (The Lancefield Bactericidal Assay) was tested using protocol described by Lancefield (1957). Briefly, overnight cultures of indicated bacterial strains were back-diluted (1:20) in 10 mL of fresh medium and incubated for 75 minutes (OD_600_ ≈ 0.15), serially diluted in PBS, and 100 μL of the 10^−4^ dilution was plated on solid medium (starting inoculum). 100 μL of the 10^−4^ dilution was mixed with 900 μL of freshly drawn, heparinized whole human blood in a heparin coated Eppendorf tube (Fisher Scientific) and incubated for 3 hours while shaking (220 rpm) at 37°C. Bacteria were next 10-fold diluted in PBS and 100 μL of undiluted and diluted suspension was plated on solid medium. The ability of GAS to survive in whole human blood is represented as multiplication factor (M. F.), which was calculated using following formula(*CFU final* /*CFU initial*). CFU final = CFU/mL after 3 hours of incubation, CFU initial = CFU in 100 μL of bacterial suspension mixed with the blood.

To determine whether RBC membrane-binding molecular mimicry affected GAS proliferation in whole human blood, overnight cultures were adjusted to a concentration 2 ^×^ 10^7^ CFU/mL, and 100 μL of bacteria were preincubated with 100 μL of either PBS or 4% RBC solution (prepared as described in the Quantitative in vitro hemolytic activity assay section; final concentration 2% RBCs) for 1 hour at 37°C. Bacteria were serially diluted in PBS and 100 μL of the suspension containing approximately 500 CFUs was plated for exact bacterial enumeration or mixed with 900 μL of fresh human blood. The following incubation and plating steps were performed as described above. The fold change between the results obtained for bacteria preincubated with RBCs and PBS within each biological replicate was calculated using the following formula: (*RBC* −*PBS* /*PBS*). In the formula, RBC = results for bacteria preincubated in 2% RBC solution and PBS = results for bacteria preincubated in PBS. Experiments were performed in three biological replicates.

#### Quantitative *In vitro* Hemolytic Activity Assay

The method for quantification of GAS strains hemolytic properties was based on previously described protocols (Saroj et al., 2016; Shin et al., 1999). Overnight cultures of M1 5448 wt pDC*erm*, Δ*ess* pDC*erm*, and Δ*ess* pDC*erm*::*ess* were back-diluted (1:20) in 10 mL of fresh medium, incubated at 37°C until the mid-exponential phase of growth (4 hours). Cultures were adjusted with THY to cell density of 10^7^ CFU/mL. A 2% red blood cell (RBC) suspension was prepared by diluting 25 μL of freshly drawn, heparinized whole human blood in 0.5 mL of PBS. The solution was subjected to centrifugation for 10 minutes at 1,000 ^×^ *g*, room temperature followed by two wash steps with equivalent volumes of PBS. RBCs were finally resuspended in 1.25 mL PBS. A 100 μL aliquot of bacteria was mixed with 100 μL of the 2% RBC suspension in an Eppendorf tube and incubated for 1 hour at 37°C. THY broth alone was used as a control for spontaneous RBCs lysis, and THY broth supplemented with Triton X-100 (1% final concentration in the mixture with RBC) (VWR International, LLC) was used to determine maximum RBCs lysis. Following incubation, tubes were centrifuged for 10 minutes at 1000 ^×^ *g* at room temperature, and 100 μL of the resulting supernatant was transferred into a flat-bottom 96-well plate (Fisher Scientific). The amount of released hemoglobin was quantified by measuring absorbance at 405 nm using a VersaMax Tunable Microplate Reader. Experiments were performed on three separate occasions in technical duplicate on each occasion.

#### Bacterial Survival in Normal Human Serum

To measure the survival of GAS strains in normal human serum (NHS), overnight cultures of M1 5448 wt pDC*erm*, Δ*ess* pDC*erm*, and Δ*ess* pDC*erm*::*ess* were back-diluted (1:20) in 10 mL of fresh medium and incubated at 37°C to the mid-exponential phase of growth (4 hours). Cultures were harvested by centrifugation for 10 minutes at 10,000 ^×^ *g* and pelleted bacteria were suspended in PBS to cells density of 10^6^ CFU/mL. Bacteria were serially diluted (10^−1^ − 10^−5^) in PBS and 5 μL spotted (in technical duplicate) on solid medium for CFU enumeration. 100 μL of bacteria (10^5^ CFU) was mixed with either 500 μL NHS (Fisher Scientific) and 400 μL PBS (final NHS concentration 50%) or 900 μL NHS (final NHS concentration 90%). Heat inactivated NHS (hNHS; 30 minutes incubation at 56°C) was used as a control. Bacterial suspensions were incubated for 3 hours at 37°C, serially diluted in PBS and spotted (in technical duplicates) on solid medium for CFU enumeration as described above. Bacterial survival was calculated using the formula (*CFU final* /*CFU initial*)×100, where CFU final = average CFU/mL after 3 hours of incubation, CFU initial = average CFU in 100 μL of bacterial suspension mixed with the NHS. Experiments were performed on three separate occasions.

#### THP-1-Derived Macrophage Infection

The procedure for GAS infection of THP-1-derived macrophages was based on a previously-described protocol (O’Neill et al., 2016), with several modifications. THP-1 cells were differentiated into macrophages by harvesting the desired amount of cells by centrifugation (6 minutes at 150 ^×^ *g*, room temperature), and suspending them in complete growth medium supplemented with 25 nM PMA. Cells (10^5^) were seeded in 24-well plate (Fisher Scientific) and incubated for 48 hours at 37°C in the presence of 5% CO_2_. Overnight cultures of indicated GAS strains were centrifuged for 10 minutes at 10,000 ^×^ *g* at room temperature and suspended in RPMI 1640 Medium (ATCC modification) supplemented with 10% heat inactivated normal human serum and 0.05 mM 2-mercaptoethanol to a final concentration of 2 ^×^ 10^5^ CFU/mL. Bacterial cells were opsonized by incubation for 20 minutes at room temperature. Differentiated THP-1 cells were washed once with PBS and 1 mL of the bacterial suspension was added (multiplicity of infection [MOI] of 2). Following 90 minutes of infection at 37°C, extracellular bacteria were killed by adding gentamycin (VWR International, LLC) to a final concentration of 150 μg/mL. At the same time, medium with bacteria from one set of wells was serially diluted (10^−1^ − 10^−2^) in PBS and the rest of the medium was removed. Macrophages were washed three times with PBS, then lysed by adding 1 mL of 0.05% Triton X-100 in PBS solution and incubating for 5 minutes at 37°C. The solution was 10-fold diluted in PBS and 5 μL of diluted medium and lysed macrophages were spotted on solid medium for CFU enumeration in technical duplicate. After 1 hour of gentamycin treatment at 37°C, the medium was removed, cells were washed three times with PBS, and fresh medium (RPMI 1640 Medium [ATCC modification], 10% heat inactivated normal human serum, 0.05 mM 2-mercaptoethanol) supplemented with 150 μg/mL gentamycin was added. Cells from one set of wells were lysed, as described above, and 10 μL was spotted on solid medium for bacterial enumeration in technical triplicate. Macrophages were incubated for 6 hours at 37°C. At time points 2, 4, and 6 hours cells were lysed as described above and 10 μL was spotted on solid medium for bacteria enumeration in technical triplicate. The following formula was used to calculate the amount of bacteria associated with macrophages: (*T*90 *Cells* /*T*90 *Cells* + *T*90 *Medium*)×100. The amount of internalized bacteria was calculated using the following formula: (*T*1*G* /*T*90 *Cells* + *T*90 *Medium*)×100. Bacterial survival within macrophages at indicated time points was calculated as follows: (*TX* /*T*1*G*)×100. Abbreviations in the above formulas are as follows: T90 Cells = average CFU/mL from lysed macrophages after 90 minutes of infection (bacteria that were adhered to and internalized by macrophages), T90 Medium = average CFU/mL in medium after 90 minutes of infection, T1G = average CFU/mL in lysed macrophages after 1 hour of gentamycin treatment, TX – average CFU/mL in lysed macrophages at indicated time points following 1 hour of gentamycin treatment. Experiments were performed on three separate occasions.

To determine whether RBC membrane-binding molecular mimicry affected the interactions between GAS and THP-1 derived macrophages, overnight cultures were adjusted to a concentration 2 ^×^ 10^7^ CFU/mL, and 100 μL of bacteria were preincubated with 100 μL of either PBS or 4% RBC solution (prepared as described in the Quantitative *in vitro* hemolytic activity assay section; final concentration 2% RBCs). 100 μL of 4% RBC solution was incubated with 100 μL of THY broth or THY broth with Triton X-100 (1% final concentration) as a control for RBC lysis. Following 1 hour incubation at 37°C, bacterial cells were serially diluted (10^−1^ − 10^−4^) in PBS. 5 μL of each dilution was spotted onto solid medium for CFU scoring, and undiluted bacteria were harvested by centrifuged for 10 minutes at 10,000 ^×^ *g*. After centrifugation, supernatants were collected and used for the quantitative *in vitro* hemolytic activity assay (described in the above section) and cells were suspended in RPMI 1640 Medium (ATCC modification) supplemented with 10% heat inactivated normal human serum and 0.05 mM 2-mercaptoethanol to a final concentration of 2 ^×^ 10^5^ CFU/mL. The following steps were performed as described above. The fold change between the results obtained for bacteria preincubated with RBCs and PBS within each biological replicate of the experiment was calculated using the following formula: (*RBC* −*PBS* /*PBS*). In the formula, RBC = results for bacteria preincubated in 2% RBC solution and PBS = results for bacteria preincubated in PBS. Experiments were performed in three biological replicates.

#### Human Neutrophil Extracellular Killing

Whole blood was isolated from healthy donors into heparinized vacutainer tubes (Becton Dickinson). Blood was layered onto Polymorphprep (Progen) and subjected to centrifugation at 500 × *g* for 30 minutes in a swing-bucket rotor at room temperature without braking. The neutrophil layer was extracted and washed once in 10 mL of Hank’s Buffered Salt Solution (HBSS) (GIBCO) and spun for 5 minutes at 400 × *g*. The cell pellet was resuspended in a solution of RPMI (Sigma) supplemented with 10% heat-killed normal human serum (Millipore). 1 × 10^6^ cells/mL were added to 24 well plates for neutrophil killing studies. Bacteria were opsonized in RPMI supplemented in 10% heat killed normal human serum prior to addition to neutrophil suspension studies. For extracellular killing studies, bacteria were added to cells at MOI of 1 and incubated at 37°C for 45 minutes. Following incubation, cells were resuspended and the entire volume of the well was subjected to centrifugation at 400 × *g* for 5 minutes. Supernatants were subjected to serial dilution from 10^−1^ to 10^−2^ and 20 μL was spotted onto THY+2 μg/mL erythromycin agar plates for CFU enumeration. Recovered extracellular CFU following incubation with primary neutrophils was calculated using the following formula: Log_2_(*Recovered CFU* /*mL* /*Inoculated CFU* /*mL*).

#### Human Neutrophil Intracellular Uptake

Neutrophils were isolated as described above. Bacteria were incubated with neutrophils at MOI of 2 for 45 minutes at 37°C. Extracellular bacteria were killed through addition of 100 μg/mL Gentamycin for 30 minutes at 37°C. Neutrophils were resuspended and subjected to centrifugation at 400 × *g* for 5 minutes. Cell pellets were lysed in 100 μL of sterile ultrapure water, diluted from 10° to 10^−1^, and 20 μL was spotted onto agar plates for CFU enumeration. Recovered intracellular CFU following incubation with primary neutrophils was calculated using the following formula: Log_2_(*Recovered CFU* /*mL* /*Inoculated CFU* /*mL*).

#### Hyaluronic Acid Capsule Quantification

The amount of hyaluronic acid capsule produced by tested GAS strains was det*erm*ined as described previously (Jin and Pancholi, 2006). Briefly, a standard curve for det*erm*ining hyaluronic acid capsule formation was prepared. This was achieved by mixing 50 μL of hyaluronic acid sodium salt from *Streptococcus equi* (Sigma Aldrich) 2 mL of chromogenic reagent (20 mg of Stains-all [Fisher Scientific] and 60 μL glacial acetic acid [Fisher Scientific] in 100 mL of 50% formamide [VWR International, LLC]) and measuring absorbance at 640 nm. A 10 mL overnight culture of GAS was harvested by centrifugation for 10 minutes at 10,000 ^×^ *g* at room temperature. Pelleted cells were suspended in 0.5 mL of ddH_2_O. Next, 1 mL of chloroform (Fisher Scientific) was added, bacteria were vortexed for 15 minutes, and centrifuged for 10 minutes at 12,000 ^×^ *g* at room temperature. 50 μL of the aqueous phase was collected, mixed with 2 mL of chromogenic reagent, and absorbance was measured. Hyaluronic acid capsule concentration was calculated based on the standard curve as “μg/mL.” Experiments were performed in biological triplicate.

#### RBCNS Binding by GAS Cells

Mouse Red Blood Cell (RBC) membrane nanosponges (RBCNS) were prepared as described previously (Hu et al., 2015; Lapek et al., 2017a). Briefly, 100 nm PLGA polymeric cores were prepared using 0.67 dL/g of Carboxyl t*erm*inated 50:50 poly(lactic-co-glycolic) acid (PLGA) (LACTEL Absorbable Polymers) in a nanoprecipitation process. The PLGA polymer was first dissolved in acetone at a concentration of 10 mg/mL. One milliliter of the solution was then added to 1 mL of UltraPure water. For fluorescently labeled formulations, 1,1’-Dioctadecyl-3,3,3′,3′-Tetramethylindodicarbocyanine Perchlorate (DiD) (Life Technologies Corporation) was loaded into the polymeric cores at 0.1 wt%. The mixture was next stirred under vacuum for 3 hours. RBC membrane coating was obtained by fusing RBC membrane vesicles with PLGA particles via sonication using an FS30D bath sonicator at a frequency of 42 kHz and a power of 100 W for 2 min. The size and the zeta-potential of the resulting RBCNS were obtained from three dynamic light scattering measurements using a Malvern ZEN 3600 Zetasizer, which showed an average hydrodynamic diameter of 100 nm and 115 nm before and after the membrane coating process, respectively.

To determine binding of RBCNS to GAS cells, 2 mL of the indicated strain overnight culture was mixed in 1:1 ratio with mixture of 1 part VECTASHIELD Mounting Medium with DAPI (Fisher Scientific) and 9 parts 10% sucrose or with 10% sucrose, 4% bovine serum albumin (BSA) (VWR International, LLC) and incubated for 30 minutes at room temperature. Solutions were next centrifuged for 8 minutes at 3,000 ^×^ *g*, room temperature and washed twice with 10% sucrose. Pelleted bacteria were suspended in 10% sucrose (original culture volume), placed on ice and incubated for 5 min with or without RBCNS. Afterward, 1 mL of 10% sucrose was added and samples were centrifuged for 8 minutes at 3,000 ^×^ *g*, room temperature, washed three times, and suspended in 10% sucrose. A TECAN plate reader was used to measure fluoresce intensity of DiD at excitation/emission 630/670 nm and DAPI at excitation/emission 358/461 nm. Measurements of DAPI-free and RBCNS-free bacteria were used to determine the fluorescent background. The RBCNS binding by bacterial cells was calculated using formula (NS /*DAPI* –*B*), where NS = DiD fluorescence signal (excitation/emission 630/670 nm); DAPI = DAPI fluorescence signal (excitation/emission 358/461 nm); B - fluorescence signal of DAPI- and RBCNS-free bacterial cells (excitation/emission 358/461 nm). Experiments were performed in biological triplicate.

#### α-S Protein Bacterial Blocking

Bacteria were cultured overnight and diluted to 10^7^ CFU/mL concentration. Bacteria were subjected to centrifugation at 10,000 × *g* for 10 minutes at room temperature. Bacteria were resuspended in 1 mL 1× sterile PBS and subjected to centrifugation at 10,000 × *g* for 10 minutes at room temperature. The wash step was repeated once more. Washed bacteria were suspended in 1 mL of RPMI 1640 medium supplemented with 10% heat inactivated normal human serum, incubated for 20 minutes shaking at room temperature, washed with PBS as above, and suspended in 1 mL of RPMI 1640. Serum collected from rabbits on the pre-immunization bleed day and the final post-S protein immunization day were incubated at 56°C for 20 minutes and added to bacterial suspensions at 1:100 dilutions. Bacteria were incubated shaking for 40 minutes at room temperature. Bacteria were pelleted at 10,000 × *g* for 10 minutes, supernatants removed, and pellets were resuspended in 200 μL of THY broth. For RBC binding studies, 100 μL of each bacterial suspension was incubated with 100 μL of RBCs prepared as described above. Mixtures were incubated at 37°C for 1 hour, then pelleted at 10,000 × g for 10 minutes at room temperature. Complete RBC lysis was ensured by measuring heme release as described above. Bacteria were resuspended in 800 μL of sterile 0.75× PBS. Bacteria were pelleted, imaged, and resuspended prior to serial titration and plating on THY+2 μg/mL erythromycin plates for enumeration.

#### Measurement of Δess pDCerm::emm1 Cell Sedimentation

The effect of elevated M protein expression in the Δ*ess* genetic background on cell aggregation and sedimentation was analyzed as follows. Bacterial strains were inoculated in the morning in 10 mL of liquid medium, incubated at 37°C for 8 hours, back-diluted (1:20) in 10 mL of fresh medium (in a 15 mL falcon tube), and incubated for 18 hours at 37°C. On the following day, tubes were spun down (1 minute, 500 ^×^ *g*, room temperature) to bring down all aggregated cells to the bottom of the tube. Sedimented cell pellet heights and top diameters were measured with a millimeter scale ruler and non-sediment culture OD_600_ values were determined. Cultures were next vortexed, serially diluted (10^−1^ − 10^−5^) in PBS. 5 μL of each sample was spotted on solid medium for CFU/mL scoring in technical duplicate. Sedimented bacterial volume (mm^3^) was calculated using the volume equation for a circular truncated cone:$(1/3)\pi \left(r_{1}^{2}+r_{1}r_{2}+r_{2}^{2}\right)h$; where r_1_ = radius of the top of the sedimented cells; r_2_ = radius of the bottom of the sediment cells (constant value of 2 mm); h = height of the sedimented cells. Experiments were performed in three biological replicates.

#### Mouse Systemic Infection Model

To determine the fitn*ess* of GAS strains during mouse systemic infection, overnight cultures of indicated bacterial strains were back-diluted (1:20) in 10 mL of fresh medium and incubated at 37°C for 4 hours to mid-exponential phase of growth. Cultures were next harvested (10 minutes centrifugation at 10,000 ^×^ *g*, room temperature) and pelleted bacteria were suspended in PBS to cell density of 10^8^ CFU/mL. Bacteria were serially diluted (10^−1^ − 10^−6^) in PBS and 5 μL spotted (in technical duplicates) on solid medium for exact CFU enumeration. The 6–8 week old female CR (CD-1) mice (10 per group) were infected with 100 μL of bacterial suspensions (10^7^ CFU) via lateral tail vein injection. Control animals were injected with PBS alone. Animal survival and weight (g) was monitored daily for 10 days. Change in body weight was calculated with the following formula: (W× /W0)×100, where W0 = animal weight at the day of the infection and W× = animal weight at indicated day.

To determine whether RBC membrane-binding molecular mimicry affected GAS virulence during mouse systemic infection, overnight cultures of wt pDC*erm* strains was back-diluted (1:20) in 10 mL of fresh medium, incubated at 37°C for 4 hours, mixed with equal volumes of PBS or 4% mouse RBC solution (prepared as described in the Quantitative in vitro hemolytic activity assay section), and incubated for 1 hour at 37°C. The subsequent steps were performed as described above.

For analysis of bacterial load in the blood and organs of infected animals, bacterial suspensions were prepared and administered to animals (7 per group) as described above. At day 4 post-infection, blood was collected, animals were sacrificed and perfused with PBS (administered through the apex of the left ventricle of the heart). Organs (liver, kidneys, spleen, lungs, and heart) were next removed, submerged in 1 mL of PBS, and homogenized for 1 minute in Mini-Beadbeater-24 (BioSpec Products) with Ceramic Beads (BioSpec Products). Blood and homogenized organs were next serially diluted in (10^−1^ − 10^−6^) in PBS and 10 μL of undiluted or 5 μL diluted blood/tissue was spotted on solid medium (with or without supplementation of erythromycin) for bacteria enumeration. Maintenance of the pDC*erm* vectors among bacterial strains during infection (named here “Plasmid maintenance”) was calculated by formula: (*CFU S* /*CFU NS*), where CFU S = CFU/mL of bacteria that grew on a selective medium (with erythromycin), CFU NS = CFU/mL of bacteria that grew on a non-selective medium (without erythromycin). In situation where no bacteria were recovered on one of the media types, a 10-fold lower CFU/mL value of the other media type was assigned to it.

For analysis of bacterial load in the blood and spleens through the initial 4 days of infection and spleen collection for proteomic analysis, bacterial suspensions were prepared and administered to animals (5 per group for each day of the study) as described above. Control animals were mock-infected with PBS. During the subsequent 4 days post-infection, weight measurements of animals were taken before blood collection and spleen harvesting (performed as described above). Prior to tissue homogenization, spleen weight measurements were taken. Following plating (on solid media without erythromycin) for the bacterial CFU/mL determination, homogenized spleens were immediately transferred to −80°C. Spleen weight as a percent of the total body mass was calculated with following formula: (*SW* /*BW*)×100, where SW = spleen weight and BW = weight of the entire animal.

For studies involving *Ifnar1* −/− mice, mice were infected with 10^7^ CFU of Δ*ess* GAS or administered PBS via lateral tail vein injection (n = 8 per group). Survival was monitored for 3 weeks following injection.

#### Protection Studies with Δ*ess* Strain

In order to test if mice exposed to systemic infection with Δ*ess* developed adaptive immunity against the wt pDC*erm* strain, overnight cultures of the mutant strain were back-diluted (1:20) in 10 mL of fresh medium and incubated at 37°C to mid-exponential phase of growth (4 hours of growth). Cultures were centrifuged for 10 minutes at 10,000 ^×^ *g* at room temperature, and cells were suspended in PBS to a concentration of 3 ^×^ 10^8^ CFU/mL. Bacteria were serially diluted (10^−1^ − 10^−6^) in PBS and 5 μL spotted on solid medium for exact CFU determination in technical duplicate. A group of 20 CR (CD-1) mice (6–8 week old) were administered 100 μL of a bacterial suspension containing approximately 3 ^×^ 10^7^ CFU through lateral tail vein injection. 20 control animals were injected with PBS. After 3 weeks, half of the mice from each group were infected with 5 ^×^ 10^7^ CFUs of wt pDC*erm* (bacteria suspension was prepared as described above), and the second half of each group was mock-infected with PBS. Animal survival and weight (g) were monitored daily for 10 days. Changes in body weight were calculated as described in the above section. At the end of day 10 post-infection, blood from surviving animals was collected into heparin-coated Eppendorf tube for serum isolation.

#### SDS-PAGE and Western Blotting

Bacterial samples from liquid cultures were obtained by separating cells from culture supernatants by 10 minutes centrifugation at 10,000 ^×^ *g* at room temperature. Samples were normalized based on the total protein concentration or optical density (OD_600_) of bacterial culture and boiled for 10 minutes in 4× Laemmli Sample Buffer (Bio-Rad Laboratories) containing 50 mM Dithiothreitol (DTT) (Invitrogen). Samples were separated on either 10% or 15% polyacrylamide gels. Following electrophoresis, proteins were stained with InstantBlue (Expedeon) or transferred onto 0.2 μm nitrocellulose membrane (Bio-Rad Laboratories) using a Trans-blot Turbo (Bio-Rad Laboratories) system. Membranes were incubated in 5% milk suspension in PBS with 0.1% Tween 20 (VWR International, LLC) (PBST) for 1 hour, briefly washed with PBST, incubated for 1 hour in primary antibodies diluted in a 5% milk suspension in PBST, washed 3 times (10 minutes each) with PBST, incubated with secondary HRP conjugate antibodies for 1 hour, and finally washed 3 times (8 minutes each) with PBST. Immunoblots were incubated for 5 minutes in SignalFire ECL Reagent (Cell Signaling Technology, Inc.) mix and visualized with Chemi-DocTM MP System (Bio-Rad Laboratories). Primary antibodies were used at indicated dilutions: rabbit polyclonal anti-S protein 1:5000, mouse polyclonal anti-M protein 1:1000, pooled mouse serum from experimental groups 1:5000. Secondary antibodies, Goat Anti-Rabbit IgG H&L (HRP) and Goat F(ab) Anti-Mouse IgG H&L (HRP) (Abcam, Inc.), were used at 1:10,000 dilution.

#### Proteomics Analyses

Samples of whole cells and culture supernatants of wild-type, Δ*ess*, and complemented GAS M1 5448 were collected by centrifugation of overnight cultures (10 mL) for 10 minutes at 10,000 ^×^ *g* at 4°C. A quarter of cOmplete, Mini, EDTA-free Protease Inhibitor Cocktail tablet was added immediately to each filter sterilized (0.22 μm) culture supernatant and samples were stored −80°C until used. Bacterial cells and culture supernatants were collected on three separate occasions.

Sample lysis was performed as follows. Bacterial cell pellets were suspended in 500 μL of lysis buffer composed of 75 mM NaCl, 3% sodium dodecyl sulfate (SDS) (Fisher Scientific), 1 mM sodium Fluoride (VWR International, LLC), 1 mM beta-glycerophosphate (Sigma Aldrich), 1 mM sodium orthovanadate, 10 mM sodium pyrophosphate (VWR International, LLC), 1 mM phenylmethylsulfonyl fluoride (Fisher Scientific), 50 mM HEPES (Fisher Scientific) pH 8.5, and 1X cOmplete EDTA-free protease inhibitor cocktail, plus 500 μL of 8M Urea (Fisher Scientific), 50 mM HEPES pH 8.5, and subjected to sonication using a Q500 QSonica sonicator (Qsonica) equipped with 1.6 mm microtip at amplitude 20%. Samples were subjected to 10 s of sonication followed by 10 s of rest, with a total sonication time of 30 s. For mouse spleens, 500 μL of lysis buffer and 500 μL of 8M Urea, 50 mM HEPES pH 8.5 was added to homogenized samples in PBS. In order lyse any remaining intact tissue, spleens were subjected to an additional three rounds of 1 minute bead beating in a Mini-Beadbeater-24 at 4°C with 1 minute of rest between cycles. For immunoprecipitated antigens, the lysis step was omitted, and only 210 μL of 8M Urea, 50 mM HEPES pH 8.5 was added.

Protein extraction and digestion was performed as follows. Reduction of disulfide bonds was performed by addition of DTT to a final concentration of 5 mM. Samples were incubated for 30 minutes at 56°C. Iodoacetamide (IAA) (Sigma Aldrich) was added to a final concentration of 15 mM and samples were incubated in a darkened environment for 20 minutes at room temperature. The reaction was quenched by adding 5mM DTT and incubated in a darkened environment for 15 minutes at room temperature. Proteins from spleen samples and bacterial culture supernatants were precipitated using chloroform-methanol precipitation (Wessel and Flügge, 1984). Briefly, protein solutions were mixed with 6 mL of methanol, 1.5 mL of chloroform, and 4 mL of HPLC-grade water. Samples were briefly vortexed and centrifuged for 2 minutes at 4,000 rpm at room temperature. The resulting supernatants were aspirated and an additional 6 mL of methanol was added to the pellets. Samples were briefly vortexed and centrifuged for 2 minutes at 4,000 rpm. Supernatants were again aspirated. Protein pellets were placed on ice and washed three times with 3 mL of ice cold acetone (Fisher Scientific) (briefly vortexing and 2 minutes spin at 4,000 rpm at 4°C). Proteins from bacterial cell pellets and immunoprecipitated antigens were precipitated by adding 1/4 of the total volume of trichloroacetic acid (TCA) (Fisher Scientific) to the samples, briefly vortexing the samples, and incubating samples on ice for 10 minutes. Samples were spun down for 5 minutes at 16,000 ^×^ *g* at 4°C. Protein pellets were washed three times with 300 μL of ice cold acetone. The chloroform-methanol- and TCA-precipitated proteins were dried at 56°C.

Protein Digestion and Tandem Mass Tag (TMT)-Labeling was performed as follows. Dried bacterial protein pellets (from whole cells, culture supernatants, and immunoprecipitated antigens) were suspended in 300 μL of digestion buffer comprised of 1M urea and 50mM HEPES pH 8.5, while dried mouse spleen proteins were suspended in 900 μL of the same buffer. Samples were vortexed for 5 minutes and sonicated in a water bath for 5 minutes. Bacterial and mouse proteins were digested by adding 3 or 9 μg of LysC Endopeptidase (VWR International, LLC), respectively, and shaking overnight at room temperature. Following day, 3 or 8.6 μg of Sequencing Grade Modified Trypsin (Core Bio Services) was added to bacterial or mouse proteins, respectively, and samples were incubated for 6 hours at 37°C. The digestion reactions of bacterial, mouse, and immunoprecipitation samples was terminated by acidifying the solution with 20 μL, 60 μL of 10% trifluoroacetic acid (Sigma Aldrich), or 20 μL of 10% trifluoroacetic acid plus 300 μL 0.1% of trifluoroacetic acid, respectively. Insoluble debris was separated by centrifuging the samples for 5 minutes at 16,000 ^×^ *g* at room temperature. Supernatants containing digested soluble peptides of bacterial whole cell lysates and culture supernatants, and mouse splenic tissues were desalted using C18 resin columns (Sepax). Peptides from immunoprecipitated antigens were desalted using a STAGE (STop And Go Extraction) TIPS Desalting Procedure, and dried under vacuum. All samples, with the exception of the immunoprecipitated antigens, were next suspended in a solution of 50% acetonitrile (VWR International, LLC) and 5% formic acid (Fisher Scientific) and peptide content was quantified with Pierce Quantitative Colorimetric Peptide Assay (Fisher Scientific). 50 μg of peptides from each sample was separated for further analysis, with the exception of the bacterial supernatants samples, which displayed chronically low yields. Therefore, 37 μg from bacterial supernatant samples were separated for further study. Internal standard bridge channels were prepared for mouse splenic tissues and bacterial whole cell samples using methods previously described (Lapek et al., 2017b). Briefly, 5.83 μg of each sample was mixed together and separated into seven 50 μg aliquots. Internal standards were dried under vacuum. The immunoprecipitated antigens were suspended in 50 μL of 30% dry acetonitrile with 200 mM HEPES pH 8.5 and the entire sample was used for TMT-labeling. TMT reagents were prepared by vortexing for 5 minutes in a solution of 30% dry acetonitrile with 200 mM HEPES pH 8.5 to a final concentration of 20 μg/μL. Label assignment was performed such that no sample replicates were assigned to the same label. Bridge channels were assigned to channel 126 for spleen and whole cell bacteria experiments. Protein aliquots were incubated with 8 μL of suspended TMT labels for 1 hour at room temperature. Reaction quenching was performed by adding 9 μL of 5% hydroxylamine (Sigma Aldrich) to the labeling reaction and incubating for 15 minutes at room temperature. Following the reaction quenching, samples were acidified with 50 μL of 1% trifluoroacetic acid. Following labeling, samples within each 10-plex were pooled, and with exception to immunoprecipitated antigens, desalted, and lyophilized (McAlister et al., 2014; Thompson et al., 2003; Wang et al., 2011).

Reverse-Phase High pH Liquid Chromatography Sample Fractionation of bacterial and mice TMT-labeled samples was performed as follows. Lyophilized multiplexed samples were suspended in a solution of 5% acetonitrile, 5% formic acid and fractionated using reverse-phase high pH liquid chromatography on an Ultimate 3000 HPLC with 4.6 mm × 250 mm C18 column. Samples were separated on a gradient progressing from 5% to 90% acetonitrile in 10 mM ammonium bicarbonate (Fisher Scientific) for one hour. For labeled and pooled immunoprecipitated antigens, Pierce High pH Reversed-Phase Peptide Fractionation Kit (Fisher Scientific) was used for sample fractionation. Of the 96 and 8 fractions produced for bacteria/mice and immunoprecipitated antigens samples, respectively; concatenated fractions were pooled as previously described (Wang et al., 2011). Alternating pooled sets were lyophilized under vacuum.

LC-MS/MS was performed as follows. Dried fractions were suspended in 8 μL of 5% acetonitrile and 5% formic acid solution, vortexed for 5 minutes, and sonicated in a water bath for 5 minutes. Experiments were performed on an Orbitrap Fusion mass spectrometer with in-line Easy-nLC. Fractions were run on three-hour gradients beginning with elution solution containing 3% acetonitrile and 0.125% formic acid and ending with an elution solution containing 100% acetonitrile and 0.125% formic acid. Peptides were separated using an in house-packed 30 cm ^×^ 100 μm inner diameter, 360 μm outer diameter column comprised of 0.5 cm C4 resin (diameter = 5 μm), 0.5 cm C18 resin (diameter = 3 μm), and 29 cm C18 resin (diameter = 1.8 μm). Source ionization was performed by applying 2000V of electricity through the T-junction joining sample, waste, and column capillary termini.

MS1 spectrum acquisition was performed in data-dependent mode with Orbitrap survey scan range of 500 – 1200 *m/z* and resolution of 60,000. Automatic gain control (AGC) 2 ^×^ 10^5^ with maximum ion inject time of 100 ms. Top N was used with N = 10 for both MS2 and MS3 fragment ion analysis.

MS2 data were collected using the decision tree option. Ions carrying 2 charges were analyzed between 600 – 1200 *m/z*, and ions carrying 3 or 4 charges were analyzed between 500 – 1200 *m/z*. The ion intensity threshold was 5 ^×^ 10^4^. Selected ions were isolated in the quadrupole at 0.5 Th and fragmented via Collision Induced Dissociation (CID). Fragment ion detection and data centroiding occurred in the linear ion trap with rapid scan rate AGC target of 1 ^×^ 10^4^.

TMT-based quantitation via MS3 fragmentation was performed using synchronous precursor selection. Up to 10 MS2 precursors were concurrently fragmented using High Energy Collisional Dissociation (HCD) fragmentation. Reporter ions were detected in the Orbitrap at a resolution of 60,000 and with a lower threshold of 110 *m/z*. AGC was set to 1 ^×^ 10^5^ with maximum ion inject time of 100 ms. Data collected was centroided and precursor ions outside of 40 *m/z* below and 15 *m/z* above the MS2 *m/z* were removed.

Data Processing and Normalization was performed as follows. Raw spectral data were processed using Proteome Discoverer 2.1. Spectral matching at the MS2 level was performed against the UniProt *Streptococcus pyogenes serotype M1* reference proteome downloaded on 1/29/2018 or *Mus musculus* reference proteome downloaded on 11/28/2016. The Sequest algorithm was used for spectral matching and *in silico* decoy database construction (Eng et al., 1994). Precursor ion mass tolerance was set to 50 ppm and fragment ion mass tolerance was set to 0.6 Da. The digesting enzyme was specified as trypsin, with two missed cleavages allowed and peptide length range between 6 – 144 amino acids. Methionine oxidation was used as dynamic modification (+15.995 Da). Used static modifications included isobaric tandem mass tags at peptide N-termini and on lysine residues (+229.163 Da) and carbamidomethylation of cysteine residues (+57.021 Da). A false discovery rate of 1% was used for filtering at both the peptide and protein levels in Percolator using the aforementioned decoy database (Käll et al., 2007; Spivak et al., 2009).

The single pooled bridge channel assigned to TMT-126 was used for normalization of values across 10-plexes for the spleen tissues and whole cell lysate bacterial proteomics experiments, while the supernatants proteomics data were normalized against average values for each protein. Bridge channels had been eschewed for the bacterial supernatants proteomics experiment due to a low protein yield following precipitation. Briefly, data were filtered for high peptide spectral match (PSM) confidence and PSM unambiguity/selection. Quantification values were normalized against protein bridge channel values or protein average value and subsequently to the median value of all bridge channels or averages. The resulting quantification values were next adjusted for variation in labeling efficiency by normalizing against the median values for each TMT label and the median of the channel medians. To adjust the data for an observed 10-plex-based batch effect, the frozen surrogate variable analysis (fSVA) R package was used (Parker et al., 2014).

### QUANTIFICATION AND STATISTICAL ANALYSIS

All data, excepting proteomics datasets, were analyzed and plotted using GraphPad Prism 7. Mean values with corresponding ± SEM are presented; Kaplan-Meier survival curves were prepared for Figures 4A, S3A, and 6A. GraphPad Prism’s built-in Student’s t test and one way ANOVA were used to determine statistical significance (p < 0.05).

For bacterial whole cells and culture supernatants proteomic data statistical significance (p < 0.05) was determined by using Microsoft Excel’s built-in Student’s t test with Welch’s Correction where appropriate based on an antecedent F test. Further stringency was imposed on binary comparisons through the use of the pi score calculation, which accounts for both statistical significance and fold change (Xiao et al., 2014). A pi score cutoff of 1.1082 was used to identify differentially expressed proteins between the various GAS strains profiled. Annotation of significantly altered proteins was performed manually. Treemaps were generated using the treemap R package.

For mouse spleen proteomic data, unbiased clustering was performed using the amap R package. Distance measurement was performed using the Spearman method and the average agglomeration method was used for clustering. Dendrogram generation was performed using the ape R package. Heatmap generation was performed using the gplots R package. STEM clustering was performed using the Short Time series Expression Miner (STEM) software package (Ernst and Bar-Joseph, 2006). Eight clusters were generated using the average values for each treatment group time series. For protein interaction network analysis, protein lists generated using STEM clustering were subjected to analysis using String-db (https://string-db.org/cgi/input.pl). Interactions were filtered for aggregate scores greater than 0.7 (high confidence). Network figures were generated using Cytoscape v3.7.1. Fold difference was calculated by dividing wild-type and Δ*ess* abundance values by the average of the PBS values, then calculating Log_2_ of the wild-type versus Δ*ess* values. P values were calculated using the PBS-normalized values.

### DATA AND CODE AVAILABILITY

The mass spectrometry proteomics data have been deposited into MassIVE (https://massive.ucsd.edu/ProteoSAFe/static/massive.jsp) and submitted to the ProteomeXchange Consortium (http://proteomecentral.proteomexchange.org) with the dataset identifiers PXD015341 for bacterial proteomics, PXD015342 for supernatant proteomics, and PXD015343 for spleen proteomics.

## Supplementary Material

1

2

3

4

5

6

## Figures and Tables

**KEY RESOURCES TABLE:** 

REAGENT or RESOURCE	SOURCE	IDENTIFIER
Antibodies		
Rabbit polyclonal Anti-S protein	This work	N/A
Mouse polyclonal Anti-M1 protein	Kindly provided by Dr. Victor Nizet (UCSD) (Hollands et al., 2010)	N/A
Mouse antibodies against various *S. pyogenes* M1 5448 protein (pooled serum obtained from 4 animal experimental groups)	This work	N/A
Goat Anti-Rabbit IgG H&L (HRP)	Abcam, Inc.	Cat#ab6721; RRID: AB_955447
Goat F(ab) Anti-Mouse IgG H&L (HRP)	Abcam, Inc.	Cat#ab6823; RRID: AB_955395
Bacterial and Virus Strains		
*Escherichia coli* NEB 5-alpha	New England BioLabs	Cat#C2987I
*Escherichia coli* NEB Turbo	New England BioLabs	Cat#C2984I
*Escherichia coli* NEB Turbo pHY304	This work	N/A
*Escherichia coli* NEB Turbo pHY304-*ess*::*cat*	This work	N/A
*Escherichia coli* NEB 5-alpha pDC*erm*	This work	N/A
*Escherichia coli* NEB 5-alpha pD*Cerm*::*ess*	This work	N/A
*Escherichia coli* NEB Turbo pD*Cerm*::*emm1*	This work	N/A
*Escherichia coli* BL21(DE3)	New England BioLabs	Cat# C2527I
*Escherichia coli BL21*(DE3) N-HisPP-pET-28a(+)::*ess*	This work	N/A
*Streptococcus pyogenes* M1 5448	Private laboratory stock; originally provided by Dr. Victor Nizet (UCSD) (Chatellier et al., 2000)	N/A
*Streptococcus pyogenes* M1 5448 pHY304-*ess*::*cat*	This work	N/A
*Streptococcus pyogenes* M1 5448 Δ*ess*	This work	N/A
*Streptococcus pyogenes* M1 5448 pDC*erm*	This work	N/A
*Streptococcus pyogenes* M1 5448 Δ*ess* pDC*erm*	This work	N/A
*Streptococcus pyogenes* M1 5448 Δ*ess* pDC*erm*::*ess*	This work	N/A
*Streptococcus pyogenes* M1 5448 Δ*ess* pDC*erm*::*emm1*	This work	N/A
Chemicals, Peptides, and Recombinant Proteins		
Todd Hewitt Broth	Spectrum Laboratory Products, Inc.	Cat#743–29433-12
Yeast Extract	VWR International, LLC	Cat#90000–026 (EA)
Agar Granulated	VWR International, LLC	Cat#90000–782 (EA)
Mueller Hinton Agar w/5% Sheep Blood	Fisher Scientific	Cat#R04055
Erythromycin	Spectrum Laboratory Products, Inc.	Cat# TCI-E0751–25G
Difco LB Agar, Miller (Luria-Bertani)	Spectrum Laboratory Products, Inc.	Cat#743–29229-10
LB Agar	Core Bio Services	Cat#C121
Kanamycin sulfate	BioPioneer	Cat#C0031
Glycerol	VWR International, LLC	Cat#IC19520491 (EA)
RPMI 1640 Medium (ATCC modification)	Life Technologies Corporation	Cat#A1049101
Fetal Bovine Serum (USDA Certified, Heat Inactivated)	Core Bio Services	Cat#FB-02
GIBCO 2-Mercaptoethanol	Life Technologies Corporation	Cat#21985023
Phorbol 12-myristate 13-acetate	Sigma Aldrich	Cat#P8139–10MG
Q5® High-Fidelity DNA Polymerase	New England BioLabs	Cat# M0491L
Deoxynucleotide (dNTP) Solution Mix	New England BioLabs	Cat#N0447S
EagI-HF	New England BioLabs	Cat#R3505S
HindIII-HF	New England BioLabs	Cat#R3104S
SOC Outgrowth Medium	New England BioLabs	Cat#B9020S
Quick-Load® Taq 2X Master Mix	New England BioLabs	Cat#M0271L
Glycine Anhydride	Spectrum Laboratory Products, Inc.	Cat#G3063–100GM
Sucrose	Sigma Aldrich	Cat# RDD023–1KG
Magnesium Chloride Hexahydrate, MgCl_2_ ⋅ 6H_2_O	Fisher Scientific	Cat#ICN19469880
Phosphate Buffered Saline (PBS-20X)	Cell Signaling Technology	Cat#9808S
KpnI-HF	New England BioLabs	Cat#R3142S
BamHI-HF	New England BioLabs	Cat#R3136T
Isopropyl β-D-1-thiogalactopyranoside (IPTG)	Omega Scientific	Cat#IP-05
Tris(Base)	Avantor Performance Materials	Cat#JT4109–2
Imidazole	Sigma Aldrich	Cat#792527–500G
L-Arginine, 98+%	VWR International, LLC	Cat# AAA15738–14
L-Glutamic Acid	Spectrum Laboratory Products, Inc.	Cat#G1036–1KG
Triton X-100 Polyethylene Glycol p-tert-Octylphenyl Ether	Fisher Scientific	Cat#BP151100
Lysozyme	BioPioneer	Cat#C0021
Ni-NTA Agarose	QIAGEN	Cat#30210
Precision Plus Protein Unstained Standards	Bio-Rad Laboratories	Cat#1610363
InstantBlue	Core Bio Services	Cat# ISB1L
FM4–64 Dye (N -(3-Triethylammoniumpropyl)-4-(6-(4-(Diethylamino) Phenyl) Hexatrienyl) Pyridinium Dibromide)	Life Technologies Corporation	Cat#T3166
DAPI	Sigma Aldrich	Cat#D9542–10MG
SYTOX Green Nucleic Acid Stain - 5 mM Solution in DMSO	Life Technologies Corporation	Cat#S7020
Agarose (Broad Separation Range for DNA/RNA/Genetic Analysis Grade)	Fisher Scientific	Cat#BP1356500
Ammonium sulfate, Enzyme grade, ≥ 99%, (NH_4_)_2_SO_4_	Fisher Scientific	Cat#ICN15037380
Potassium Phosphate Dibasic Trihydrate ≥ 99%, K_2_HPO_4_ ⋅ 3H_2_O	Fisher Scientific	Cat#ICN19484580
Potassium Phosphate Monobasic, 99+%, KH_2_PO_4_	Fisher Scientific	Cat#ICN19472790
Sodium citrate dehydrate, 99%, Trisodium citrate dihydrate, C_6_H_5_O_7_Na_3_ ⋅ 2H_2_O	Fisher Scientific	Cat#ICN19486894
Methanol, ≥ 99.9%, Methyl Alcohol, CH_4_O	Fisher Scientific	Cat#A4524
Urea	Fisher Scientific	Cat#U15500 (CS)
Magnesium sulfate heptahydrate, MgSO_4_ ⋅ 7H_2_O	Sigma Aldrich	Cat#M2773–500G
*n*-Hexadecane, CH_3_(CH_2_)_14_CH_3_	Fisher Scientific	Cat#AAA10322AE
Triton® X-100	VWR International, LLC	Cat#IC807423 (EA)
Carboxyl terminated 50:50 poly(lactic-co-glycolic) acid (PLGA)	LACTEL Absorbable Polymers	Cat#B6013–2
DiD’ oil; DiIĈ18^^^(5) oil (1,1’-Dioctadecyl-3,3,3′, 3′-Tetramethylindodicarbocyanine Perchlorate)	Life Technologies Corporation	Cat#D307
VECTASHIELD Mounting Medium with DAPI	Fisher Scientific	Cat# NC9524612
Bovine Serum Albumin (BSA)	VWR International, LLC	Cat#IC810032 (EA)
Normal Human Serum	Fisher Scientific	Cat#5058826
Gentamicin, 10 mg/mL	VWR International, LLC	Cat#10128–220 (EA)
Hyaluronic acid sodium salt from *Streptococcus equi*	Sigma Aldrich	Cat#53747–1G
Stains-all, 97%, 1-Ethyl-2-[3-(1-ethylnaphtho [1, 2-d]thiazolin-2-ylidene)-2-methylpropenyl] naphtho[1, 2-d]thiazolium bromide, C_30_H_27_BrN_2_S_2_	Fisher Scientific	Cat#AC213510010
Acetic Acid, Glacial, CH_3_COOH	Fisher Scientific	Cat#MKV155500
Formamide	VWR International, LLC	Cat#PI17899 (EA)
Chloroform	Fisher Scientific	Cat#C6061
EDTA 0.5M PH8.0	Fisher Scientific	Cat#50983251
Protein A/G Agarose Max Flow, Highly Cross-linked Beads, 4%	Genesee Scientific Corporation	Cat#20–540
EGTA	Fisher Scientific	Cat#507516812
4× Laemmli Sample Buffer	Bio-Rad Laboratories	Cat#1610747
UltraPure Dithiothreitol (DTT)	Invitrogen	Cat#15508013
Tween 20 Detergent	VWR International, LLC	Cat#80503–492 (EA)
SignalFire(tm) ECL Reagent	Cell Signaling Technology, Inc.	Cat#6883S
cOmplete, Mini, EDTA-free Protease Inhibitor Cocktail Tablets	Roche Diagnostics	Cat#11836170001
Sodium Chloride	Sigma Aldrich	Cat#S7653–250G
Sodium Dodecyl Sulfate (SDS)	Fisher Scientific	Cat#BP8200–500
Sodium Fluoride	VWR International, LLC	Cat#JT3688–1 (EA)
beta-Glycerophosphate disodium salt hydrate	Sigma Aldrich	Cat#G5422–500G
Sodium Vanadate Sodium Orthovanadate, Na_3_O_4_V	Fisher Scientific	Cat#S454–50
Sodium Pyrophosphate Decahydrate	VWR International, LLC	Cat#JT3850–1 (EA)
Phenylmethylsulfonyl Fluoride PMSF, C_7_H_7_FO_2_S	Fisher Scientific	Cat#71105GM
HEPES > = 99% N-(2-Hydroxyethyl)piperazine- N-2-ethanesulfonic Acid, C_8_H_18_N_2_O_4_S	Fisher Scientific	Cat#BP310500
Iodoacetamide (IAA)	Sigma Aldrich	Cat#I1149–5G
Trichloroacetic Acid > = 99.0% TCA, C_2_HCl_3_O_2_	Fisher Scientific	Cat#A322500
Acetone 99.5% min by GC 2-Propanone, C_3_H_6_O	Fisher Scientific	Cat#A949SK1
LysC Endopeptidase, Mass Spectrometry Grade, Wako	VWR International, LLC	Cat#100369–826 (EA)
V5113 Sequencing Grade Modified Trypsin	Core Bio Services	Cat#V5113
TMT10plex Isobaric Label Reagent Set	Fisher Scientific	Cat#PI90110
Acetonitrile, Anhydrous	Fisher Scientific	Cat#NC9077861
Hydroxylamine solution	Sigma Aldrich	Cat#467804–50ML
Trifluoroacetic Acid	Sigma Aldrich	Cat#299537–100G
Acetonitrile for HPLC	VWR International, LLC	Cat#BJAH015–4PC (CS)
Formic Acid, 88% Methanoic Acid, CH_2_O_2_	Fisher Scientific	Cat#A118P500
Ammonium bicarbonate	Fisher Scientific	Cat#09830–1KG
Critical Commercial Assays		
Wizard Genomic DNA Purification Kit	VWR International, LLC	Cat#PAA1120 (EA)
QIAquick PCR Purification Kit (250)	QIAGEN, Inc.	Cat#28106
NEBuilder HiFi DNA Assembly Master Mix - 10 rxns	New England BioLabs	Cat#E2621S
QIAGEN Plasmid Mini Kit (100)	QIAGEN, Inc.	Cat#12123
Quick Ligation Kit	New England BioLabs	Cat#M2200S
DC Protein Assay	Bio-Rad Laboratories	Cat#5000116
Dual-Glo Luciferase Assay System	Promega	Cat#E2920
Quantitative Colorimetric Peptide Assay	Fisher Scientific	Cat#PI23275
Pierce High pH Reversed-Phase Peptide Fractionation Kit	Fisher Scientific	Cat#PI84868
Deposited Data		
Bacterial Proteomics Data	ProteomeXchange	PXD015341
Supernatant Proteomics Data	ProteomeXchange	PXD015342
Spleen Temporal Infection Proteomics Data	ProteomeXchange	PXD015343
Experimental Models: Cell Lines		
THP-1	ATCC	Cat#TIB-202
Experimental Models: Organisms/Strains		
Mouse: ICR (CD-1) Outbred Mice	Envigo	RRID:IMSR_CRL:22
Mouse: *Ifnar1* −/− in C57BL/6 Background	MMRRC	32045-JAX
Oligonucleotides		
Primers used in this study are listed in the Table S5	Integrated DNA Technologies, Inc.	N/A
Recombinant DNA		
pACYC184	Kindly provided by Dr. Victor Nizet (UCSD) (Nakano et al., 1995)	N/A
pHY304	Kindly provided by Dr. Victor Nizet (UCSD) (Pritzlaff et al., 2001)	N/A
pHY304-*ess*::*cat*	This work	N/A
PDC*erm*	Kindly provided by Dr. Victor Nizet (UCSD) (Jeng et al., 2003)	N/A
pDC*erm*::*ess*	This work	N/A
N-HisPP-pET-28a(+)	Kindly provided by Dr. Partho Ghosh (UCSD)(Buffalo et al., 2016)	N/A
N-HisPP-pET-28(+)::*ess*	This work	N/A
pDC*erm*::*ermm1*	This work	N/A
Software and Algorithms		
NEBuilder Assembly Tool	New England BioLabs	http://nebuilder.neb.com/
BPROM	Softberry	http://www.softberry.com/berry.phtml?topic=bprom&group=programs&subgroup=gfindb
Promoter Prediction by Neural Network	Martin Reese	http://www.fruitfly.org/seq_tools/promoter.html
Clustal Omega	Sievers et al., 2011	https://www.ebi.ac.uk/Tools/msa/clustalo/
PRALINE	Simossis and Heringa, 2005	http://ibi.vu.nl/programs/pralinewww/
Phyre2	Kelley et al., 2015	http://www.sbg.bio.ic.ac.uk/phyre2/html/page.cgi?id=index
SoftWoRx v5.5.1	Applied Precisionin	N/A
FIJI	SciJava	https://fiji.sc
CellProfiler	Kamentsky et al., 2011	https://cellprofiler.org/
Proteome Discover	Thermo-Fisher	N/A
R Studio	RStudio Team (2015). RStudio: Integrated Development for R. RStudio, Inc., Boston, MA URLhttps://rstudio.com/	https://rstudio.com/
fSVA package	Parker et al., 2014	N/A
Short Time-series Expression Miner (STEM)	Ernst and Bar-Joseph, 2006	http://www.cs.cmu.edu/jernst/stem/
String-db	String Consortium 2019	https://string-db.org/cgi/input.pl
Cytoscape v3.7.1	National Resource for Network Biology	https://www.cytoscape.org
GraphPad Prism	GraphPad Prism version 7.00 for Windows, GraphPad Software, La Jolla California USA,https://www.graphpad.com	https://www.graphpad.com

## Highlights

- S protein promotes red blood cell binding to group A Streptococcus (GAS) cell surface
- Red blood cell membrane binding is an immune evasion tactic used by GAS
- Lack of S protein results in a drastic reduction of virulence during systemic infection
- Attenuated virulence during **Δ***ess* infection is associated with altered immunity

## References

[R1] Allen RC, Popat R, Diggle SP, and Brown SP (2014). Targeting virulence: can we make evolution-proof drugs? Nat. Rev. Microbiol 12, 300–308. 24625893 10.1038/nrmicro3232

[R2] Baron C (2010). Antivirulence drugs to target bacterial secretion systems. Curr. Opin. Microbiol 13, 100–105. 20079679 10.1016/j.mib.2009.12.003

[R3] Bronte V, and Pittet MJ (2013). The spleen in local and systemic regulation of immunity. Immunity 39, 806–818. 24238338 10.1016/j.immuni.2013.10.010 PMC3912742

[R4] Brook I (2013). Penicillin failure in the treatment of streptococcal pharyngotonsillitis. Curr. Infect. Dis. Rep 15, 232–235. 23588893 10.1007/s11908-013-0338-0

[R5] Buffalo CZ, Bahn-Suh AJ, Hirakis SP, Biswas T, Amaro RE, Nizet V, and Ghosh P (2016). Conserved patterns hidden within group A Streptococcus M protein hypervariability recognize human C4b-binding protein. Nat. Microbiol 1, 16155. 27595425 10.1038/nmicrobiol.2016.155 PMC5014329

[R6] Carapetis JR, Steer AC, Mulholland EK, and Weber M (2005). The global burden of group A streptococcal diseases. Lancet Infect. Dis 5, 685–694. 16253886 10.1016/S1473-3099(05)70267-X

[R7] Cavaillon JM, Riviere Y, Svab J, Montagnier L, and Alouf JE (1982). Induction of interferon by *Streptococcus* pyogenes extracellular products. Immunol. Lett 5, 323–326. 6819994 10.1016/0165-2478(82)90121-3

[R8] Chatellier S, Ihendyane N, Kansal RG, Khambaty F, Basma H, Norrby-Teglund A, Low DE, McGeer A, and Kotb M (2000). Genetic relatedness and superantigen expression in group A streptococcus serotype M1 isolates from patients with severe and nonsevere invasive diseases. Infect. Immun 68, 3523–3534. 10816507 10.1128/iai.68.6.3523-3534.2000 PMC97638

[R9] Churchward G, Bates C, Gusa AA, Stringer V, and Scott JR (2009). Regulation of streptokinase expression by CovR/S in Streptococcus pyogenes: CovR acts through a single high-affinity binding site. Microbiology 155, 566–575. 19202105 10.1099/mic.0.024620-0 PMC4130213

[R10] Dale JB, Batzloff MR, Cleary PP, Courtney HS, Good MF, Grandi G, Halperin S, Margarit IY, McNeil S, Pandey M, (2016). Current approaches to group A streptococcal vaccine development In Streptococcus Pyogenes: Basic Biology to Clinical Manifestations, Ferretti, Stevens, and Fischetti, eds. (University of Oklahoma Health Sciences Center), pp. 1–33. 26866216

[R11] Distler U, and Tenzer S (2017). Tools for Pathogen Proteomics: Fishing with Biomimetic Nanosponges. ACS Nano 11, 11768–11772. 29154537 10.1021/acsnano.7b07363

[R12] Dunny GM, Lee LN, and LeBlanc DJ (1991). Improved electroporation and cloning vector system for gram-positive bacteria. Appl. Environ. Microbiol 57, 1194–1201. 1905518 10.1128/aem.57.4.1194-1201.1991 PMC182867

[R13] Eng JK, McCormack AL, and Yates JR (1994). An approach to correlate tandem mass spectral data of peptides with amino acid sequences in a protein database. J. Am. Soc. Mass Spectrom 5, 976–989. 24226387 10.1016/1044-0305(94)80016-2

[R14] Ernst J, and Bar-Joseph Z (2006). STEM: a tool for the analysis of short time series gene expression data. BMC Bioinformatics 7, 191. 16597342 10.1186/1471-2105-7-191 PMC1456994

[R15] Fischetti VA (2016). M protein and other surface proteins on streptococci In Streptococcus Pyogenes: Basic Biology to Clinical Manifestations, Ferretti JJ, Stevens DL, and Fischetti VA, eds. (University of Oklahoma Health Sciences Center), p. 1 19. 26866208

[R16] Frick IM, Mörgelin M, and Björck L (2000). Virulent aggregates of Streptococcus pyogenes are generated by homophilic protein-protein interactions. Mol. Microbiol 37, 1232–1247. 10972839 10.1046/j.1365-2958.2000.02084.x

[R17] Gera K, and McIver KS (2013). Laboratory growth and maintenance of Streptococcus pyogenes (the Group A Streptococcus, GAS). Curr. Protoc. Microbiol 30, Unit9D.2. 10.1002/9780471729259.mc09d02s30 PMC3920295 24510893

[R18] Goldmann O, von Köckritz-Blickwede M, Höltje C, Chhatwal GS, Geffers R, and Medina E (2007). Transcriptome analysis of murine macrophages in response to infection with Streptococcus pyogenes reveals an unusual activation program. Infect. Immun 75, 4148–4157. 17526748 10.1128/IAI.00181-07 PMC1951976

[R19] Graham MR, Smoot LM, Migliaccio CAL, Virtaneva K, Sturdevant DE, Porcella SF, Federle MJ, Adams GJ, Scott JR, and Musser JM (2002). Virulence control in group A Streptococcus by a two-component gene regulatory system: global expression profiling and in vivo infection modeling. Proc. Natl. Acad. Sci. USA 99, 13855–13860. 12370433 10.1073/pnas.202353699 PMC129787

[R20] Gratz N, Hartweger H, Matt U, Kratochvill F, Janos M, Sigel S, Drobits B, Li X-D, Knapp S, and Kovarik P (2011). Type I interferon production induced by *Streptococcus* pyogenes-derived nucleic acids is required for host protection. PLoS Pathog. 7, e1001345. 21625574 10.1371/journal.ppat.1001345 PMC3098218

[R21] Hollands A, Pence MA, Timmer AM, Osvath SR, Turnbull L, Whitchurch CB, Walker MJ, and Nizet V (2010). Genetic switch to hypervirulence reduces colonization phenotypes of the globally disseminated group A streptococcus M1T1 clone. J Infect Dis 202, 11–19. 20507231 10.1086/653124 PMC2880657

[R22] Hu C-MJ, Fang RH, Wang K-C, Luk BT, Thamphiwatana S, Dehaini D, Nguyen P, Angsantikul P, Wen CH, Kroll AV, (2015). Nanoparticle biointerfacing by platelet membrane cloaking. Nature 526, 118–121. 26374997 10.1038/nature15373 PMC4871317

[R23] Hyland KA, Brennan R, Olmsted SB, Rojas E, Murphy E, Wang B, and Cleary PP (2009). The early interferon response of nasal-associated lymphoid tissue to *Streptococcus* pyogenes infection. FEMS Immunol. Med. Microbiol 55, 422–431. 19243434 10.1111/j.1574-695X.2009.00540.x

[R24] Jeffery CJ (1999). Moonlighting proteins. Trends Biochem. Sci 24, 8–11. 10087914 10.1016/s0968-0004(98)01335-8

[R25] Jeffery CJ (2003). Moonlighting proteins: old proteins learning new tricks. Trends Genet 19, 415–417. 12902157 10.1016/S0168-9525(03)00167-7

[R26] Jeffery CJ (2014). An introduction to protein moonlighting. Biochem. Soc. Trans 42, 1679–1683. 25399589 10.1042/BST20140226

[R27] Jeng A, Sakota V, Li Z, Datta V, Beall B, and Nizet V (2003). Molecular genetic analysis of a group A Streptococcus operon encoding serum opacity factor and a novel fibronectin-binding protein, SfbX. J. Bacteriol 185, 1208–1217. 12562790 10.1128/JB.185.4.1208-1217.2003 PMC142848

[R28] Jin H, and Pancholi V (2006). Identification and biochemical characterization of a eukaryotic-type serine/threonine kinase and its cognate phosphatase in Streptococcus pyogenes: their biological functions and substrate identification. J. Mol. Biol 357, 1351–1372. 16487973 10.1016/j.jmb.2006.01.020

[R29] Käll L, Canterbury JD, Weston J, Noble WS, and MacCoss MJ (2007). Semi-supervised learning for peptide identification from shotgun proteomics datasets. Nat. Methods 4, 923–925. 17952086 10.1038/nmeth1113

[R30] Kamentsky L, Jones TR, Fraser A, Bray M-A, Logan DJ, Madden KL, Ljosa V, Rueden C, Eliceiri KW, and Carpenter AE (2011). Improved structure, function and compatibility for CellProfiler: modular high-throughput image analysis software. Bioinformatics 27, 1179–1180. 21349861 10.1093/bioinformatics/btr095 PMC3072555

[R31] Kelley LA, Mezulis S, Yates CM, Wass MN, and Sternberg MJ (2015). The Phyre2 web portal for protein modeling, prediction and analysis. Nat Protoc. 10, 845–858. 25950237 10.1038/nprot.2015.053 PMC5298202

[R32] Lancefield RC (1957). Differentiation of group A streptococci with a common R antigen into three serological types, with special reference to the bactericidal test. J. Exp. Med 106, 525–544. 13475611 10.1084/jem.106.4.525 PMC2136803

[R33] Lapek JD Jr., Fang RH, Wei X, Li P, Wang B, Zhang L, and Gonzalez DJ (2017a). Biomimetic Virulomics for Capture and Identification of Cell-Type Specific Effector Proteins. ACS Nano 11, 11831–11838. 28892626 10.1021/acsnano.7b02650

[R34] Lapek JD Jr., Lewinski MK, Wozniak JM, Guatelli J, and Gonzalez DJ (2017b). Quantitative Temporal Viromics of an Inducible HIV-1 Model Yields Insight to Global Host Targets and Phospho-Dynamics Associated with Protein Vpr. Mol. Cell. Proteomics 16, 1447–1461. 28606917 10.1074/mcp.M116.066019 PMC5546197

[R35] Lapek JD Jr., Mills RH, Wozniak JM, Campeau A, Fang RH, Wei X, van de Groep K, Perez-Lopez A, van Sorge NM, Raffatellu M, (2018). Defining Host Responses during Systemic Bacterial Infection through Construction of a Murine Organ Proteome Atlas. Cell Syst 6, 579–592.e4. 29778837 10.1016/j.cels.2018.04.010 PMC7868092

[R36] Le Breton Y, and McIver KS (2013). Genetic manipulation of Streptococcus pyogenes (the Group A Streptococcus, GAS). Curr. Protoc. Microbiol 30, Unit9D.3. 10.1002/9780471729259.mc09d03s30 PMC3920291 24510894

[R37] Lin AE, Beasley FC, Keller N, Hollands A, Urbano R, Troemel ER, Hoffman HM, and Nizet V (2015). A group A Streptococcus ADP-ribosyl-transferase toxin stimulates a protective interleukin 1β-dependent macrophage immune response. MBio 6, e00133. 25759502 10.1128/mBio.00133-15 PMC4453525

[R38] Manetti AGO, Zingaretti C, Falugi F, Capo S, Bombaci M, Bagnoli F, Gambellini G, Bensi G, Mora M, Edwards AM, (2007). Streptococcus pyogenes pili promote pharyngeal cell adhesion and biofilm formation. Mol. Microbiol 64, 968–983. 17501921 10.1111/j.1365-2958.2007.05704.x

[R39] McAlister GC, Nusinow DP, Jedrychowski MP, Wühr M, Huttlin EL, Erickson BK, Rad R, Haas W, and Gygi SP (2014). MultiNotch MS3 enables accurate, sensitive, and multiplexed detection of differential expression across cancer cell line proteomes. Anal. Chem 86, 7150–7158. 24927332 10.1021/ac502040v PMC4215866

[R40] Metzgar D, and Zampolli A (2011). The M protein of group A *Streptococcus* is a key virulence factor and a clinically relevant strain identification marker. Virulence 2, 402–412. 21852752 10.4161/viru.2.5.16342

[R41] Miettinen M, Lehtonen A, Julkunen I, and Matikainen S (2000). Lactobacilli and Streptococci activate NF-kappa B and STAT signaling pathways in human macrophages. J. Immunol 164, 3733–3740. 10725732 10.4049/jimmunol.164.7.3733

[R42] Molloy EM, Cotter PD, Hill C, Mitchell DA, and Ross RP (2011). Streptolysin S-like virulence factors: the continuing sagA. Nat. Rev. Microbiol 9, 670–681. 21822292 10.1038/nrmicro2624 PMC3928602

[R43] Müller-Alouf H, Capron M, Alouf JE, Geoffroy C, Gerlach D, Ozegowski JH, Fitting C, and Cavaillon JM (1997). Cytokine profile of human peripheral blood mononucleated cells stimulated with a novel streptococcal super-antigen, SPEA, SPEC and group A streptococcal cells. Adv. Exp. Med. Biol 418, 929–931. 9331802 10.1007/978-1-4899-1825-3_218

[R44] Nakano Y, Yoshida Y, Yamashita Y, and Koga T (1995). Construction of a series of pACYC-derived plasmid vectors. Gene 162, 157–158. 7557406 10.1016/0378-1119(95)00320-6

[R45] O’Neill AM, Thurston TLM, and Holden DW (2016). Cytosolic replication of group A *Streptococcus* in human macrophages. MBio 7, e00020–16. 27073088 10.1128/mBio.00020-16 PMC4959517

[R46] Ofek I, Whitnack E, and Beachey EH (1983). Hydrophobic interactions of group A streptococci with hexadecane droplets. J. Bacteriol 154, 139–145. 6403501 10.1128/jb.154.1.139-145.1983 PMC217440

[R47] Parker HS, Corrada Bravo H, and Leek JT (2014). Removing batch effects for prediction problems with frozen surrogate variable analysis. PeerJ 2, e561. 25332844 10.7717/peerj.561 PMC4179553

[R48] Pritzlaff CA, Chang JC, Kuo SP, Tamura GS, Rubens CE, and Nizet V (2001). Genetic basis for the beta-haemolytic/cytolytic activity of group B Streptococcus. Mol. Microbiol 39, 236–247. 11136446 10.1046/j.1365-2958.2001.02211.x

[R49] Rasko DA, and Sperandio V (2010). Anti-virulence strategies to combat bacteria-mediated disease. Nat. Rev. Drug Discov 9, 117–128. 20081869 10.1038/nrd3013

[R50] Rivera-Hernandez T, Pandey M, Henningham A, Cole J, Choudhury B, Cork AJ, Gillen CM, Ghaffar KA, West NP, Silvestri G, (2016). Differing Efficacies of Lead Group A Streptococcal Vaccine Candidates and Full-Length M Protein in Cutaneous and Invasive Disease Models. MBio 7, e00618–16. 27302756 10.1128/mBio.00618-16 PMC4916377

[R51] Rodríguez-Ortega MJ, Norais N, Bensi G, Liberatori S, Capo S, Mora M, Scarselli M, Doro F, Ferrari G, Garaguso I, (2006). Characterization and identification of vaccine candidate proteins through analysis of the group A Streptococcus surface proteome. Nat. Biotechnol 24, 191–197. 16415855 10.1038/nbt1179

[R52] Rosenberg M, Gutnick D, and Rosenberg E (1980). Adherence of bacteria to hydrocarbons: A simple method for measuring cell-surface hydrophobicity. FEMS Microbiol. Lett 9, 29–33.

[R53] Saroj SD, Maudsdotter L, Tavares R, and Jonsson A-B (2016). Lactobacilli Interfere with *Streptococcus* pyogenes Hemolytic Activity and Adherence to Host Epithelial Cells. Front. Microbiol 7, 1176. 27524981 10.3389/fmicb.2016.01176 PMC4965460

[R54] Shin SY, Kang JH, and Hahm KS (1999). Structure-antibacterial, antitumor and hemolytic activity relationships of cecropin A-magainin 2 and cecropin A-melittin hybrid peptides. J. Pept. Res 53, 82–90. 10195445 10.1111/j.1399-3011.1999.tb01620.x

[R55] Sievers F, Wilm A, Dineen D, Gibson TJ, Karplus K, Li W, Lopez R, McWilliam H, Remmert M, Söding J, (2011). Fast, scalable generation of high-quality protein multiple sequence alignments using Clustal Omega. Moll Syst Biol 7 10.1038/msb.2011.75. PMC3261699 21988835

[R56] Simossis VA, and Heringa J (2005). PRALINE: a multiple sequence alignment toolbox that integrates homology-extended and secondary structure information. Nucleic Acids Res 33, W289–W294. 15980472 10.1093/nar/gki390 PMC1160151

[R57] Sivashankari S, and Shanmughavel P (2006). Functional annotation of hypothetical proteins—A review. Bioinformation 1, 335–338. 17597916 10.6026/97320630001335 PMC1891709

[R58] Son MS, and Taylor RK (2012). Growth and maintenance of Escherichia coli laboratory strains. Curr. Protoc. Microbiol Chapter 5, Unit 5A.4. 10.1002/9780471729259.mc05a04s27 23184597

[R59] Spivak M, Weston J, Bottou L, Käll L, and Noble WS (2009). Improvements to the percolator algorithm for Peptide identification from shotgun proteomics data sets. J. Proteome Res 8, 3737–3745. 19385687 10.1021/pr801109k PMC2710313

[R60] Sumitomo T, Nakata M, Higashino M, Terao Y, and Kawabata S (2013). Group A streptococcal cysteine protease cleaves epithelial junctions and contributes to bacterial translocation. J. Biol. Chem 288, 13317–13324. 23532847 10.1074/jbc.M113.459875 PMC3650370

[R61] Thompson A, Schäfer J, Kuhn K, Kienle S, Schwarz J, Schmidt G, Neumann T, Johnstone R, Mohammed AKA, and Hamon C (2003). Tandem mass tags: a novel quantification strategy for comparative analysis of complex protein mixtures by MS/MS. Anal. Chem 75, 1895–1904. 12713048 10.1021/ac0262560

[R62] Tylewska SK, Hjertén S, and Wadström T (1979). Contribution of M protein to the hydrophobic surface properties of *Streptococcus pyogenes*. FEMS Microbiol. Lett 6, 249–253.

[R63] Wang Y, Yang F, Gritsenko MA, Wang Y, Clauss T, Liu T, Shen Y, Monroe ME, Lopez-Ferrer D, Reno T, (2011). Reversed-phase chromatography with multiple fraction concatenation strategy for proteome profiling of human MCF10A cells. Proteomics 11, 2019–2026. 21500348 10.1002/pmic.201000722 PMC3120047

[R64] Wessel D, and Flügge UI (1984). A method for the quantitative recovery of protein in dilute solution in the presence of detergents and lipids. Anal. Biochem 138, 141–143. 6731838 10.1016/0003-2697(84)90782-6

[R65] Wessels MR, Moses AE, Goldberg JB, and DiCesare TJ (1991). Hyaluronic acid capsule is a virulence factor for mucoid group A streptococci. Proc. Natl. Acad. Sci. USA 88, 8317–8321. 1656437 10.1073/pnas.88.19.8317 PMC52499

[R66] Xiao Y, Hsiao T-H, Suresh U, Chen H-IH, Wu X, Wolf SE, and Chen Y (2014). A novel significance score for gene selection and ranking. Bioinformatics 30, 801–807. 22321699 10.1093/bioinformatics/btr671 PMC3957066

[R67] Zaman SB, Hussain MA, Nye R, Mehta V, Mamun KT, and Hossain N (2017). A review on antibiotic resistance: alarm bells are ringing. Cureus 9, e1403. 28852600 10.7759/cureus.1403 PMC5573035

