# Supplementary material for: Group A Streptococcal S Protein Utilizes Red Blood Cells as Immune Camouflage and Is a Critical Determinant for Immune Evasion
Source: Cell Rep. Author manuscript; Available in PMC 2020 Jan 9. (PMC6951797; doi:10.1016/j.celrep.2019.11.001)
Supplement: 1 [file NIHMS1545826-supplement-1.pdf]

Cell Reports, Volume *29*

Supplemental Information

Group A Streptococcal S Protein Utilizes Red Blood Cells as Immune Camouflage and Is a Critical Determinant for Immune Evasion

Igor H. Wierzbicki, Anaamika Campeau, Diana Dehaini, Maya Holay, Xiaoli Wei, Trever Greene, Man Ying, Jenna S. Sands, Anne Lamsa, Elina Zuniga, Kit Pogliano, Ronnie H. Fang, Christopher N. LaRock, Liangfang Zhang, and David J. Gonzalez

Supplemental Document

Figure S1

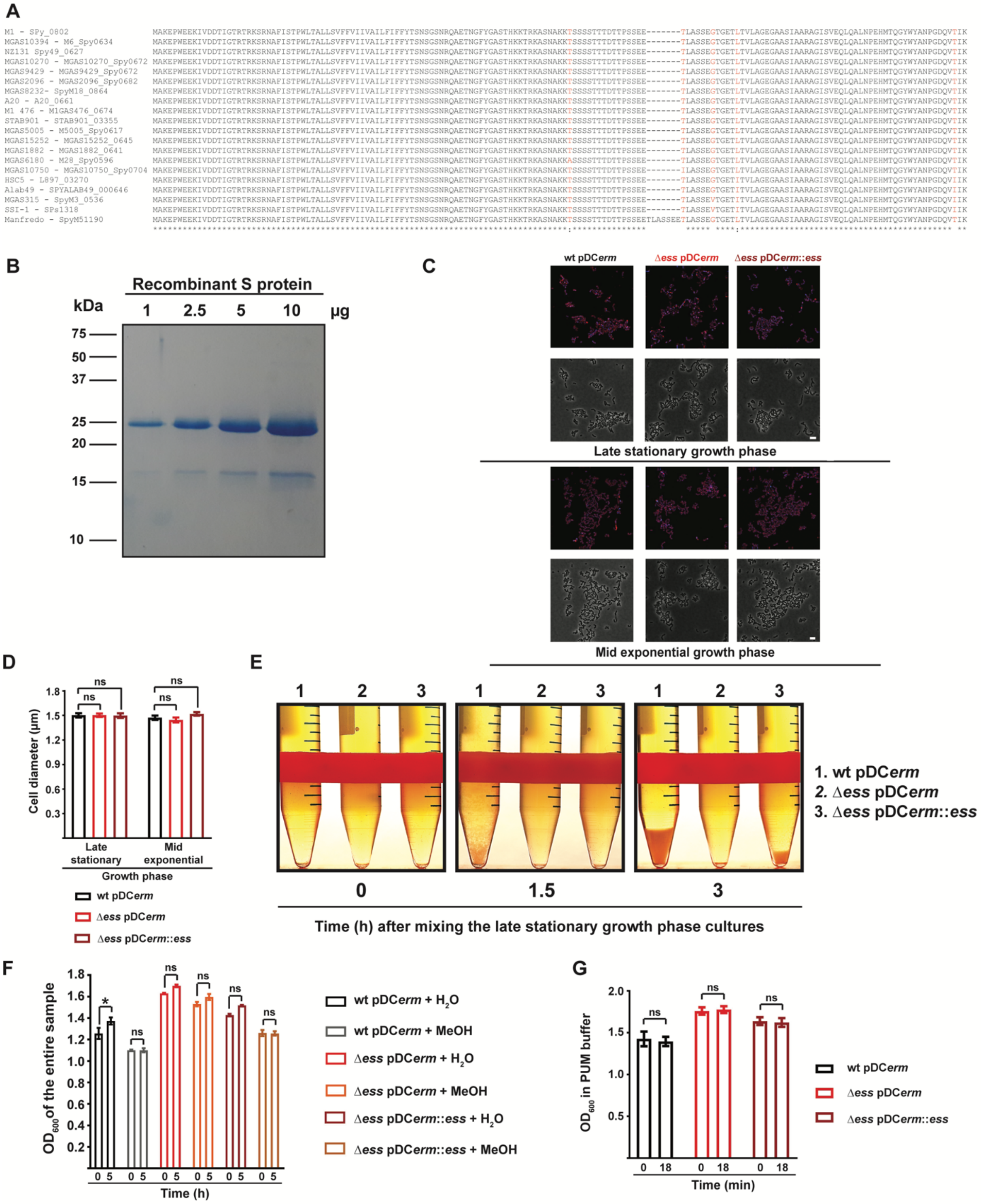

Figure S1. S protein amino acid conservation, lack of implication in physical properties of cells, and testing for spontaneous lysis during cell sedimentation and *n*-hexadecane binding experiments; Related to Figure 1

(A) Conservation of S protein at the amino acid level. Protein sequences of S protein from 20 available GAS strains from KEGG server database were aligned using Clustal Omega web tool. Variable amino acid positions are indicated in red color.

(B) Purified recombinant S protein. Indicated amounts of recombinant S protein were resolved on a 15% polyacrylamide gel and visualized by InstantBlue staining. The migration of the Precision Plus Protein Unstained Standards is indicated on the left.

(C) Fluorescent microscopy visualization of wt pDC*erm*, Δ*ess* pDC*erm*, and Δ*ess* pDC*erm*::*ess* strains late stationary and mid-exponential culture bacteria stained with FM 4-64, DAPI, and SYTOX mixture. Scale bar represents 5 μm.

(D) Quantification of bacterial average cell diameter. Cell diameters of GAS strains late stationary and mid-exponential phase of growth cultures cells were visualized by fluorescent microscopy and measured using CellProfiler software. Experiment was performed with three biological replicates. Data are represented as mean ± SEM. Statistical significance ($p < 0.05$) indicated with *.

(E) Photographic documentation of GAS strains overnight cultures cell sedimentation. Bacterial overnight cultures were vortexed and incubated at room temperature. Pictures of the cultures were taken at indicated time points.

(F) Cell intactness analysis of GAS wt pDC*erm*, Δ*ess* pDC*erm*, and Δ*ess* pDC*erm*::*ess* during 5 hours of incubation with addition of water or methanol. OD_{600} of bacterial overnight cultures mixed with either water or methanol was measured at time 0 and after mixing at time 5 hours. Experiment was performed with three biological replicates. Data are represented as mean ± SEM. Statistical significance ($p < 0.05$) indicated with *.

(G) Cell intactness analysis of GAS wt pDC*erm*, Δ*ess* pDC*erm*, and Δ*ess* pDC*erm*::*ess* during incubation in PUM buffer. OD_{600} of GAS cultures suspended in PUM buffer and not incubated with *n*-hexadecane were measured at time 0 and following 3 minutes vortexing and 15 minutes incubation (time 18 min). Experiment was performed with three biological replicates. Data are represented as mean ± SEM. Statistical significance ($p < 0.05$) indicated with *.

Figure S2

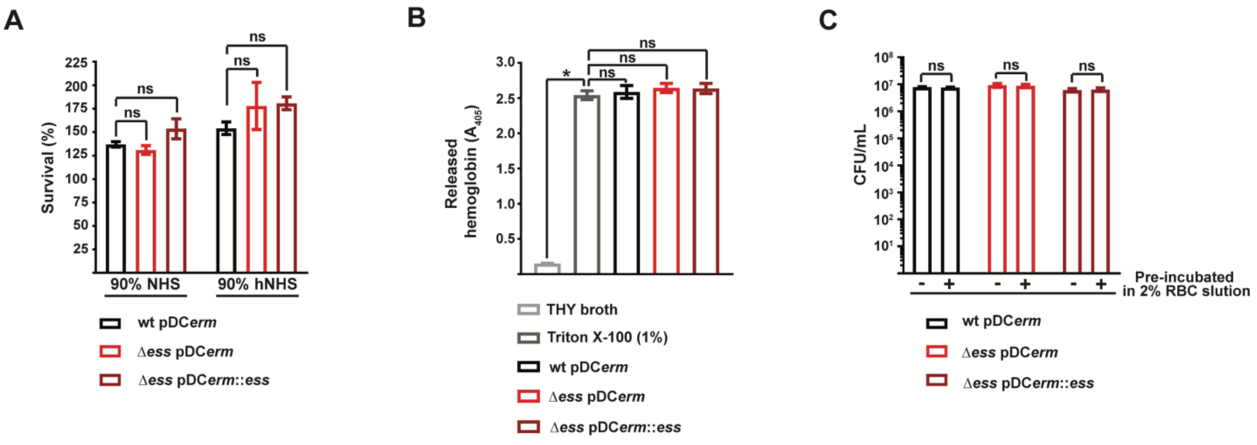

Figure S2. S protein deficient bacteria retain resistance to normal human serum killing, hemolytic properties, and their viability is not affected during incubation in 2% RBC solution. Related to Figure 2

(A) Bacterial survival in normal human serum (NHS). Resistance to serum complement killing was analyzed by incubation of GAS with 90% NHS or heat inactivated NHS (hNHS) and comparison amount of bacteria (CFUs) at time 0 and 3 hours. Experiment was performed with three biological replicates. Data are represented as mean ± SEM.

(B) Quantification of red blood cells lysis by GAS strains. Overnight cultures of wt pDC*erm*, Δ*ess* pDC*erm*, and Δ*ess* pDC*erm*::*ess* were incubated with red blood cells suspension and hemolysis was quantified by measuring hemoglobin release at absorbance 405 nm. Experiment was performed with three biological replicates. Data are represented as mean ± SEM. Statistical significance ($p < 0.05$) indicated with *.

(C) Comparison of bacteria viability after 1 hour incubation in either PBS or 2% RBC solution. Potential difference in effect of PBS [-] or 2% RBC solution [+] on viability of indicated bacterial strains was assessed by determination of bacteria amount (CFU/mL) after 1 hour of incubation. Experiment was performed with three biological replicates. Data are represented as mean ± SEM.

Figure S3

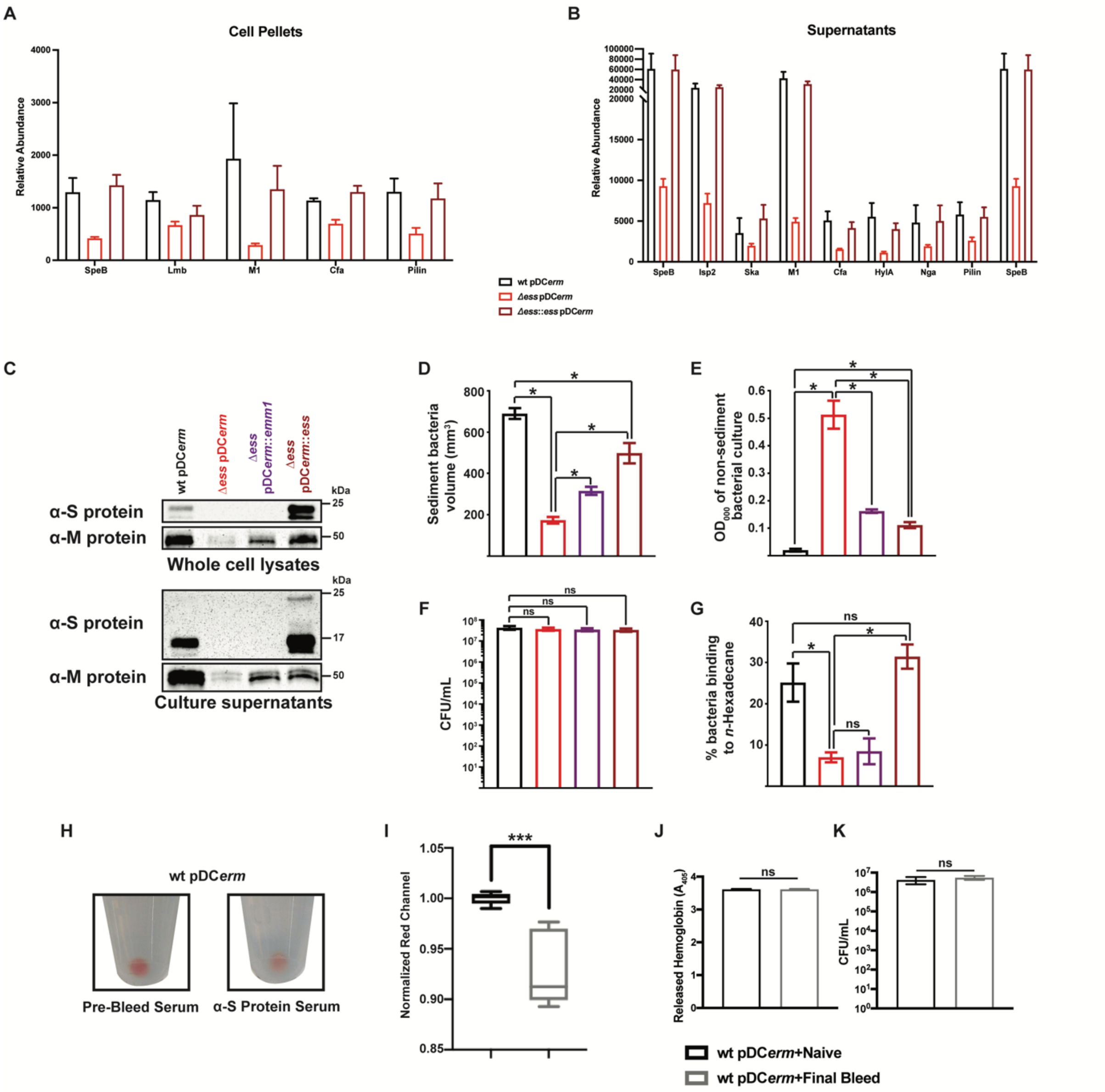

Figure S3. Distinguishing between S protein and M protein involvement in GAS cell aggregation, surface hydrophobicity, and RBC binding phenotypes. Related to Figure 3

(A) Relative protein abundance for GAS virulence factors under control of S protein in whole cell lysate proteomics. Data are represented as mean + SD.

(B) Relative protein abundance for GAS virulence factors under control of S protein in supernatant proteomics. Data are represented as mean + SD.

(C) Western blot determination of M protein abundance in Δ*ess* pDC*erm* and Δ*ess* pDC*erm*::*emm1* strain. Whole cell lysates and culture supernatants of indicated GAS strains were subjected to immunoblotting analysis with α-S protein and α-M protein antiserum.

(D) Comparison of Δ*ess* pDC*erm* and Δ*ess* pDC*erm*::*emm1* sediment bacteria volume. Volume of sediment bacteria was calculated by measuring the height and top diameter of bacterial cells on the bottom of the 15 mL falcon tube and applying the volume equation for a circular truncated cone. Data shown is representative of three biological replicates. Data are represented as mean ± SEM. Statistical significance ($p < 0.05$) indicated with *.

(E) Comparison of Δ*ess* pDC*erm* and Δ*ess* pDC*erm*::*emm1* non-sediment bacteria amount. The amount of non-sediment bacteria was determined by measuring the OD_{600} of the media above GAS cells localized on the bottom of the 15 mL falcon tube. Data shown is representative of three biological replicates. Data are represented as mean ± SEM. Statistical significance ($p < 0.05$) indicated with *.

(F) Viability of Δ*ess* pDC*erm*::*emm1* during stationary phase. Amount of live bacteria in the GAS cultures used for sedimentation studies is determined as CFU/mL. Data shown is representative of three biological replicates. Data are represented as mean ± SEM. Statistical significance ($p < 0.05$) indicated with *.

(G) Comparison of Δ*ess* pDC*erm* and Δ*ess* pDC*erm*::*emm1* cells hydrophobic properties. GAS strain hydrophobicity is determined as a percentage of culture binding to *n*-hexadecane. All experiments were performed in three biological replicates. Data shown is representative of three biological replicates. Data are represented as mean ± SEM. Statistical significance ($p < 0.05$) indicated with *.

(H) Photographic documentation of wt GAS pre-blocked with either pre-immunization inactivated rabbit serum or α-S protein immunized inactivated rabbit serum.

(I) "Redness" quantification of photographed RBC-bound GAS pellets following serum blocking and RBC incubation. Biological replicate experiments were performed on three separate days. Data shown is representative of three biological replicates. Data are represented as mean ± SEM. Statistical significance ($p<0.001$) is indicated with ***.

(J) *In vitro* hemolysis assay related to Figure S3H. Complete hemolysis was ensured by measuring A_{405} values in supernatants of each experimental replicate. Data shown is representative of three biological replicates. Data shown is representative of three biological replicates. Data are represented as mean ± SEM. Statistical significance ($p < 0.05$) indicated with *.

(K) Bacterial viability following S protein blocking assays. Bacteria were serially diluted and plated on agar plates to ensure equivalent bacterial levels following serum blocking and RBC incubation. Data shown is representative of three biological replicates. Data are represented as mean ± SEM. Statistical significance ($p < 0.05$) indicated with *.

Figure S4

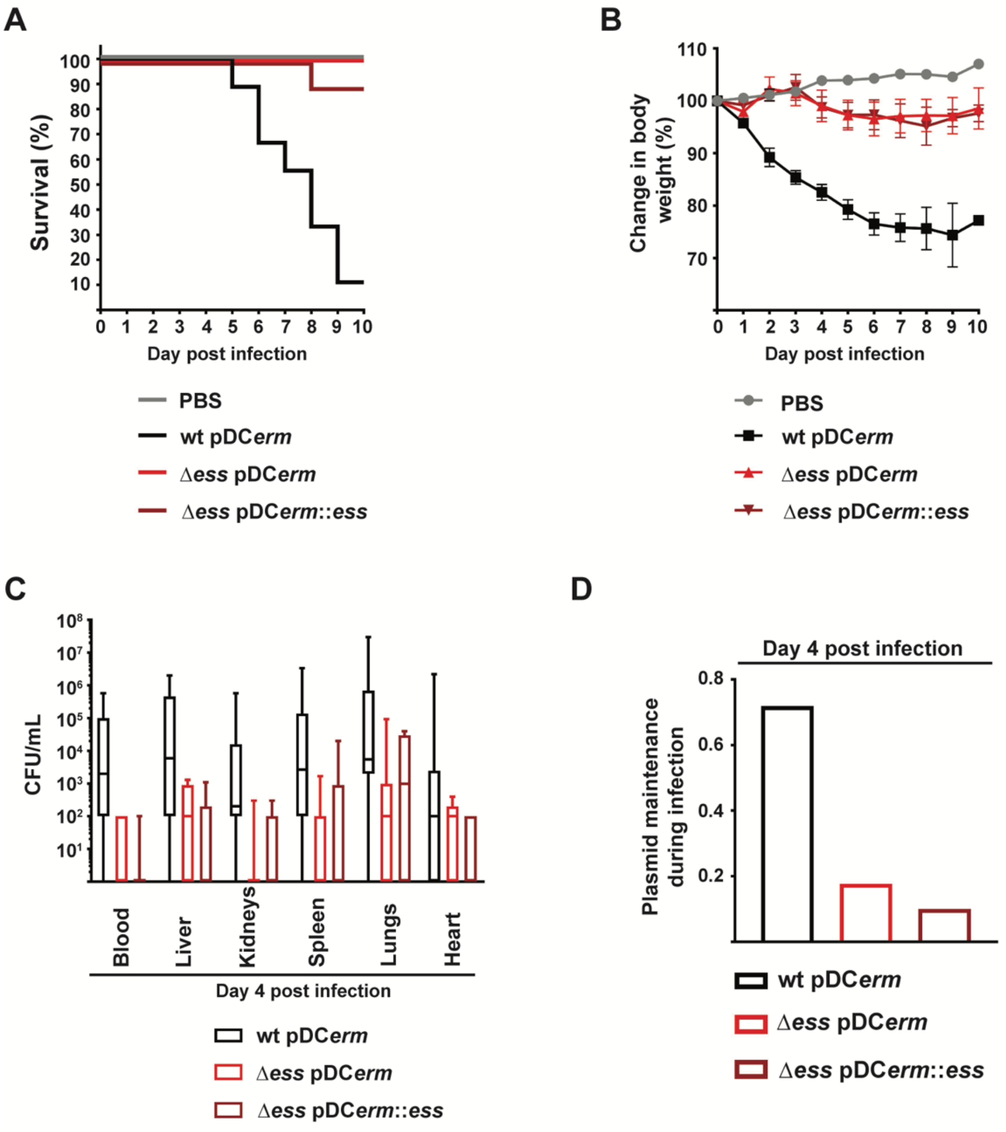

Figure S4. The Δ*ess* pDC*erm*::*ess* strain does not maintain the complementing vector during systemic infection in the mice; Related to Figure 4

(A) Survival of animals infected with wt pDC*erm*, Δ*ess* pDC*erm*, and Δ*ess* pDC*erm*::*ess* stains. Mice (n = 10) were infected intravenously with indicated bacterial strains or PBS as control and their viability was monitored over the course of 10 days. Data are presented as Kaplan-Meyer survival curves.

(B) Change in body weight of animals infected with wt pDC*erm*, Δ*ess* pDC*erm*, and Δ*ess* pDC*erm*::*ess* stains. Infected or PBS mock-infected mice (n = 10) body weight was monitored daily and change in body weight was determined by comparing weight at indicated day post infection to the weight at day 0 of the infection. Data are represented as mean ± SEM.

(C) Quantification of bacterial load in mice blood and organs during systemic infection. Bacterial burden during infection with wt pDC*erm*, Δ*ess* pDC*erm*, and Δ*ess* pDC*erm*::*ess* stains was analyzed by enumerating CFU in blood and homogenized organs (indicated in the text) collected from mice (n = 7) at day 4 post infection. Data are represented as box and whiskers plot with indicated minimum or maximum values.

(D) Plasmid maintenance during infection. Maintenance of pDC*erm* vectors among GAS stains during mouse systemic infection was determined by comparison of enumerated bacteria from plated blood and homogenized organs (indicated in the text) collected from mice (n = 7) at day 4 post infection that grew on non-selective solid medium and medium supplemented with erythromycin. Data are represented as median.

Figure S5

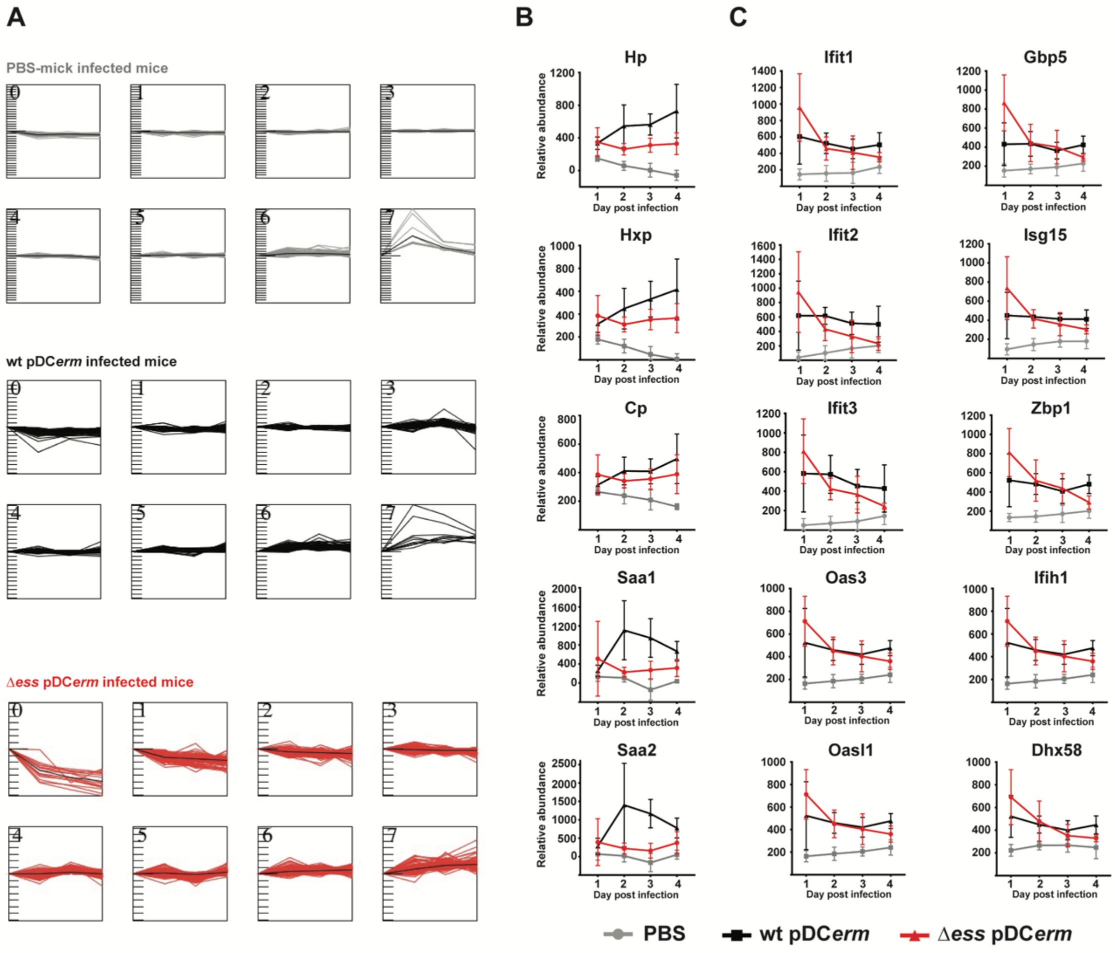

Figure S5. Data obtained from proteomic analysis of mice spleen tissues harvested from PBS mock-infected, wt pDC*erm*, and Δ*ess* pDC*erm* infected mice: STEM clustering and change in abundance of selected proteins over the course of time; Related to Figure 5

(A) STEM clustering of data obtained from proteomic analysis of mice spleen tissues harvested from PBS mock-infected, wt pDC*erm*, and Δ*ess* pDC*erm* infected mice (n=5).

(B) Change in abundance of selected acute phase proteins throughout the infection in the mice splenic tissues.

(C) Change in the abundance of interferon activated proteins throughout the infection in the mice splenic tissues.

Table S1. Excel file (Table S1.xlsx) showing mouse survival data for animals infected with wt GAS preincubated with PBS or a 2% solution of mouse RBCs (Related to Figure 2).

Table S2. Excel file (Table S2.xlsx) showing normalized quantitative proteomics results from analysis of GAS wt, Δ*ess*, and complemented bacterial cells and supernatants; tabs relate to normalized data from bacterial cells (WholeCellLysates) and supernatants (Supernatants) (Related to Figure 3).

Table S3. Excel file (Table S3.xlsx) showing normalized quantitative proteomics data from analysis of spleens harvested from mice administered PBS, wt GAS, or Δ*ess* GAS on days 1-4 post-infection (Related to Figure 5).

Table S4. Excel file (Table S4.xlsx) showing clusters from STEM analysis of quantitative proteomics results of mouse spleens on days 1-4; tabs relate to STEM clusters generated from analysis of mice administered PBS (PBS mock-infection), wt GAS (wt pDCerm infection), and Δ*ess* GAS infection (dess pDCerm infection) (Related to Figure 5).

Table S5

Oligonucleotides		
Name	Sequence 5' → 3'	Restriction enzyme recognition site
ess-up-F	AAGAATCGTATCTTTTAAATTGTTTGGTC	N/A
ess-up-R	TGATTTTTTTCTCCATGATCGTCCCCCCTTGTTAATTTAAC	N/A
cat-F	GGGGACGATCATGGAGAAAAAAATCACTGGATATACCACCGTT GATATATCCC	N/A
cat-R	TCCTCTTCAATTACGCCCCGCCCTGCCA	N/A
ess-down-F	CGGGGCGTAATTGAAGAGGAAAAAATGAAAG	N/A
ess-down-R	TAGGTTTTCTTTATAGCGG	N/A
ess-*cat*-F	GACTGATA<u>CGGCCG</u>AAGAATCGTATCTTTTAAATTG	EagI
ess-*cat*-R	ACTCGGTC<u>AAGCTT</u>TAGGTTTTCTTTATAGCGG	HindIII
pHY304-F	CTGCAGGAATTCGATATCAAGCT	N/A
pHY304-R	CCCGGGGGATCCACTAG	N/A
pHY304-Ver-F	ACACAGGAAACAGCTATGACCATG	N/A
pHY304-Ver-R	GCGCGCGTAATACGACTC	N/A
*ess*Del-Ver-F	ATAAGGTATCACTGGCAGCC	N/A
*ess*Del-Ver-F	AGGCTCTAGGACATGTCAAC	N/A
ess-F	ATCGAT<u>GGTACC</u>GGAGAAGTTATATTAAGTGG	KpnI
ess-R	TAGCCAAGATTTTTTGC	N/A
pDC*erm*-F	GCGCATGCTAAGCTTACTAG	N/A
pDC*erm*-R	CAGAACTTAAGGCGATTAAGCC	N/A
pDC*erm*-Ver-F	AGCTTTGCTAGGGGTTAC	N/A
pDC*erm*-Ver-R	CACTGCTTATTGTCAAATAGC	N/A

r*ess*-F	GGATTTACGGATCCGCTAAAGAACCATGGG	BamHI
r*ess*-R	GACTGATACGGCCGTTATTTAATAGTGACTTGATCTC	EagI
NHpET28-Ver-F	TAATACGACTCACTATAGGGG	N/A
NHpET28-Ver-R	GCGGGATATCCGGATATAG	N/A
emm1-F	ATCGATGGTACCATAGCATAAGGAGCATAAAAATGG	KpnI
emm1-R	GTTTAGTTTGTGACCTCTCC	N/A

DNA restriction enzyme recognition sites are underlined with black line

Table S5. DNA oligo sequences used for genetic manipulations of GAS strains (Related to STAR Methods).
